# Immuno-oncological effects of standard anticancer agents and commonly used concomitant drugs: an in vitro assessment

**DOI:** 10.1186/s40360-024-00746-6

**Published:** 2024-03-05

**Authors:** Tove Selvin, Malin Berglund, Lena Lenhammar, Magnus Lindskog, Malin Jarvius, Rolf Larsson, Peter Nygren, Mårten Fryknäs, Claes R Andersson

**Affiliations:** 1 Department of Medical Sciences, Division of Cancer Pharmacology and Computational Medicine, Uppsala University, SE-75185 Uppsala, Sweden; 2 Department of Immunology, Genetics and Pathology, Uppsala University, SE-75185 Uppsala, Sweden; 3 Department of Pelvic Cancer, Genitourinary Oncology Unit, Karolinska University Hospital, Stockholm, Sweden; 4 Department of Oncology-Pathology, Karolinska Institutet, Stockholm, Sweden; 5 Department of Pharmaceutical Biosciences and Science for Life Laboratory, Uppsala University, SE-751 24 Uppsala, Box 591, Sweden

**Keywords:** Anticancer drugs, Concomitant drugs, Immuno-oncology, In vitro modeling

## Abstract

**Background:**

It has become evident in the field of oncology that the outcome of medical treatment is influenced by the combined effect exerted on both cancer- and immune cells. Therefore, we evaluated potential immunological effects of 46 standard anticancer agents and 22 commonly administered concomitant non-cancer drugs.

**Methods:**

We utilized a miniaturized in vitro model system comprised of fluorescently labeled human colon and lung cancer cell lines grown as monocultures and co-cultured with activated peripheral blood mononuclear cells (PBMCs). The Bliss Independence Model was then applied to detect antagonism and synergy between the drugs and activated immune cells.

**Results:**

Among the standard anticancer agents, tyrosine kinase inhibitors (TKIs) stood out as the top inducers of both antagonism and synergy. Ruxolitinib and dasatinib emerged as the most notably antagonistic substances, exhibiting the lowest Bliss scores, whereas sorafenib was shown to synergize with activated PBMCs. Most concomitant drugs did not induce neither antagonism nor synergy. However, the statins mevastatin and simvastatin were uniquely shown to synergize with activated PBMC at all tested drug concentrations in the colon cancer model.

**Conclusion:**

We utilized a miniaturized tumor-immune model to enable time and cost-effective evaluation of a broad panel of drugs in an immuno-oncology setting in vitro. Using this approach, immunomodulatory effects exerted by TKIs and statins were identified.

**Supplementary Information:**

The online version contains supplementary material available at 10.1186/s40360-024-00746-6.

## Background

In the field of oncology, it has become evident that the treatment outcomes are influenced by the combined effect exerted on both cancer- and immune cells [1, 2]. In virtually all solid tumors, the composition of immune cells in the tumor microenvironment (TME) influences the prognosis of the patient. Conventional chemotherapeutics and targeted anticancer drugs have been shown to modulate the immune contexture and thus affect disease outcomes [3]. Some of the chemotherapeutics that are used in the clinic have severe immunosuppressive adverse effects [2] while others have the ability to enhance anti-tumor immunity [1, 4]. Furthermore, as oncology patients often suffer from cancer symptoms, treatment complications, and comorbidities, concomitant drugs such as pain killers, corticosteroids, statins, antihypertensive drugs, and antibiotics are commonly administered during the course of cancer treatment.

Herein, 46 standard anticancer drugs and 22 commonly administered concomitant drugs, selected to cover a broad range of mechanisms of actions, were evaluated using an in vitro tumor-immune model. Phenotypic screening using in vitro models that mimic the tumor immune response in order to identify novel small-molecule immunomodulators has previously been performed by us [5] and others [6]. In this study, we investigated drugs commonly used by cancer patients in such a model to identify immunological effects of potential clinical relevance and to serve as a point of reference for screens of novel compound libraries. We utilized a miniaturized model system where fluorescently labeled human cancer cells were cultured as monocultures and co-cultured with peripheral blood mononuclear cells (PBMCs). The colorectal cancer (CRC) cell line HCT116-GFP was selected as it is one of the most frequently employed cancer cell lines for evaluating anticancer agents targeting CRC; the second leading cause of cancer-related deaths worldwide, and an indication where immunotherapy is still only an option for a small subset of patients. Additionally, the widely used lung cancer cell line A549-NucLight Red (NLR) was selected to represent a diagnosis that, although being the leading cause of cancer-related deaths, is associated with a higher response rate to immunotherapy.

According to the Bliss Independence Model [7], the product of the cell viability induced by two single drugs with independent effects is expected to be equal to the cell viability induced by the combination of the two drugs. A positive Bliss score thus indicates synergy, whereas a negative Bliss score indicates antagonism. Here, the Bliss model was applied to detect synergy and antagonism between drugs and activated PBMCs. Tyrosine kinase inhibitors (TKIs) were found among the top inducers of both antagonism and synergy; ruxolitinib and dasatinib generated the lowest Bliss Scores while the multi-kinase inhibitor sorafenib was shown to synergize with activated PBMCs. Among the concomitant drugs, the immunosuppressive corticosteroids betamethasone and prednisolone had, as expected, the greatest antagonistic effects. Finally, the statins mevastatin and simvastatin were uniquely shown to synergize with activated PBMC at all tested drug concentrations. The latter results are in agreement with our previous findings demonstrating the ability of lipophilic statins to enhance immune cell-induced cancer cell death [5].

## Methods

### Cell cultures

HCT116-GFP, a human CRC cell line constitutively expressing green fluorescent protein (GFP), was obtained from AntiCancer Inc. (San Diego, CA, USA). The cells were cultured in McCoy’s 5 A medium supplemented with 10% heat-inactivated FBS, 2 mM L-glutamine, and Penicillin (100 U/mL)/ Streptomycin (100 µg/mL) (all from Sigma-Aldrich, St Louis, MO, USA). A549-NLR, a human lung cancer cell line constitutively expressing mKate2, was purchased from Essen BioScience (#4491). Cells were cultured in Ham’s F-12 Nutrient Mix, GlutaMAX, medium (Gibco #31765-027) supplemented with 10% heat-inactivated FBS, Penicillin (100 U/mL)/ Streptomycin (100 µg/mL) and Puromycin (0.5 µg/mL). Cell line authentication was performed for both cell lines by Eurofins Genomics (Ebersberg, Germany). PBMCs from three healthy, anonymous donors were isolated by Histopaque-1077 (Sigma) density gradient centrifugation and stored at -150 °C in FBS supplemented with 10% DMSO until used. All cells were cultured at 37 °C in 5% CO_2_.

### Materials

Standard anticancer drugs (Table 1) and concomitant drugs (Table 2) were purchased from LC Laboratories (MA, USA), Selleck Chemicals LLC (TX, USA), and Sigma-Aldrich. Dexamethasone was purchased from Sigma-Aldrich. All drugs were dissolved in DMSO and kept as high-concentration stock solutions at -70 °C. Recombinant human IL-2 was purchased from Peprotech (Cat# 200-02) and anti-human CD3 was purchased from ThermoFisher (Cat# 16-0037-81, RRID: AB_468854).

**Table 1:** Names of cancer drugs, drug classes, and sources of purchase

Cancer drug	Drug class	Source of purchase
Oxaliplatin	Alkylating agent	Selleck Chemicals LLC
Carboplatin	Alkylating agent	Sigma-Aldrich
Busulfan	Alkylating agent	Sigma-Aldrich
Temozolomide	Alkylating agent	Sigma-Aldrich
Melphalan	Alkylating agent	Sigma-Aldrich
Bendamustine	Alkylating agent	Selleck Chemicals LLC
Vincristine	Microtubule inhibitor	Selleck Chemicals LLC
Vinorelbine	Microtubule inhibitor	Selleck Chemicals LLC
Paclitaxel	Microtubule inhibitor	Sigma-Aldrich
Etoposide	Topoisomerase inhibitor	Sigma-Aldrich
Irinotecan	Topoisomerase inhibitor	LC Laboratories
Daunorubicin	Anthracycline	Selleck Chemicals LLC
Doxorubicin	Anthracycline	LC Laboratories
Epirubicin	Anthracycline	Sigma-Aldrich
Idarubicin	Anthracycline	Selleck Chemicals LLC
Mitoxantrone	Anthracycline	Selleck Chemicals LLC
Mitomycin	Anthracycline	Selleck Chemicals LLC
Dactinomycin	Anthracycline	Sigma-Aldrich
Thioguanine	Antimetabolite	Sigma-Aldrich
Gemcitabine	Antimetabolite	LC Laboratories
Fluorouracil	Antimetabolite	Sigma-Aldrich
5-azacytidine	Antimetabolite	Sigma-Aldrich
Gefitinib	Kinase inhibitor	LC Laboratories
Erlotinib	Kinase inhibitor	LC Laboratories
Dasatinib	Kinase inhibitor	LC Laboratories
Imatinib	Kinase inhibitor	LC Laboratories
Ponatinib	Kinase inhibitor	LC Laboratories
Regorafenib	Kinase inhibitor	Selleck Chemicals LLC
Sorafenib	Kinase inhibitor	LC Laboratories
Sunitinib	Kinase inhibitor	LC Laboratories
Alectinib	Kinase inhibitor	Selleck Chemicals LLC
Ruxolitinib	Kinase inhibitor	LC Laboratories
Palbociclib	Kinase inhibitor	LC Laboratories
Vemurafenib	Kinase inhibitor	Selleck Chemicals LLC
Temsirolimus	mTOR-inhibitor	Sigma-Aldrich
Everolimus	mTOR-inhibitor	LC Laboratories
Sirolimus	mTOR-inhibitor	LC Laboratories
Estradiol	Hormone treatment	Sigma-Aldrich
Fulvestrant	Hormone treatment	Sigma-Aldrich
Tamoxifen	Hormone treatment	Sigma-Aldrich
Octreotide	Hormone treatment	Selleck Chemicals LLC
Venetoclax	BCL-2 inhibitor	LC Laboratories
Vorinostat	HDAC inhibitor	LC Laboratories
Amsacrine	Acridine	Sigma-Aldrich
Olaparib	PARP inhibitor	LC Laboratories
Verapamil	Calcium-channel blocker	Sigma-Aldrich

**Table 2:** Names of concomitant drugs, drug classes, and sources of purchase

Concomitant drug	Drug class	Source of purchase
Paracetamol	Analgesic, antipyretic	Sigma-Aldrich
Ibuprofen	NSAID	Sigma-Aldrich
Acetylsalicylic acid	NSAID	Sigma-Aldrich
Celecoxib	NSAID	LC Laboratories
Simvastatin	Statin	Sigma-Aldrich
Mevastatin	Statin	Sigma-Aldrich
Metformin	Blood glucose lowering	Selleck Chemicals LLC
Enalapril	ACE inhibitor	Selleck Chemicals LLC
Metoprolol	Beta blocker	Selleck Chemicals LLC
Metoclopramide	Antiemetic	Selleck Chemicals LLC
Betamethasone	Corticosteroid	Selleck Chemicals LLC
Prednisolone	Corticosteroid	Sigma-Aldrich
Doxazosin	Antihypertensive	Sigma-Aldrich
Loratadine	Antihistamine	Selleck Chemicals LLC
Mycophenolate	Immune suppressor	Sigma-Aldrich
Amoxicillin	Antibiotic	Sigma-Aldrich
Piperacillin	Antibiotic	Sigma-Aldrich
Tazobactam	Antibiotic	Selleck Chemicals LLC
Sertraline	SSRI	Sigma-Aldrich
Haloperidol	Antiemetic	Sigma-Aldrich
Morphine	Opioid	Sigma-Aldrich
Omeprazole	Proton-pump inhibitor	Sigma-Aldrich

### Assessment of drug effects

Cancer cell monocultures were established by seeding HCT116-GFP (1,000 cells/50µL/well) or A549-NLR (500 cells/50µL/well) in 384-well Corning plates (for image-based analysis) or in 384-well NUNC plates (for fluorometric microculture cytotoxicity assay (FMCA)). After 24 h preincubation, PBMCs were added at a 1:4 cancer cell:PBMC ratio to establish co-cultures. Additionally, PBMCs alone (40,000 cells/50µL/well) were seeded in 384-well NUNC plates to obtain PBMC monocultures. Activation of the PBMCs was performed by supplementing the media with anti-CD3 (final concentration 100 ng/mL) and IL2 (final concentration 10 ng/mL) at the time of seeding. Anticancer drugs and concomitant drugs were added to a source plate as 10mM stock solutions and were then added to the cell plates (final concentrations 1, 10, and 30 µM) using an Echo Liquid Handler 550 (Beckman Coulter, CA, USA). Treated cell plates were placed in the Live-Cell Analysis System IncuCyte S3 (Essen Bioscience, MI, USA) and cancer cell viability was indirectly monitored by image-based quantification of GFP or mKate2 every 4 h for a total of 72 h. Additionally, cell viability in monocultures were measured using the FMCA [8]. Briefly, the cells were incubated with fluorescein diacetate (FDA) for 50 min, generating fluorescein signals in cells with intact plasma membranes. The fluorescein signals were measured using a CLARIOstar microplate reader (BMG Labtech, Germany).

### Drug-immune interaction analysis

We adapted the Bliss Independence Model which states that the product of the reduced cell viability induced by two single drugs with independent effects is expected to be equal to the reduced cell viability induced by the combination of the two drugs. Defining a Bliss score as B = (Viability Drug 1) x (Viability Drug 2) - (Viability Drug 1 + 2), a positive Bliss score thus indicates synergy while a negative Bliss score indicates antagonism. Herein, the Bliss model was used to identify synergy and antagonism between drugs and aCD3/IL-2 activated PBMCs, i.e., B = (cancer cell viability with drug alone) x (cancer cell viability with PBMCs alone) - (cancer cell viability with drug + PBMCs combined).

### Statistics

Assay validity and variability between PBMC donors were evaluated using the concordance correlation coefficient (CCC) [9, 10] which was calculated in Microsoft Excel along with confidence intervals based on the Z-transform implementation in the R-package DescTools [11]. All other statistical analysis was performed in GraphPad Prism v.9.4.0 (453). Error bars denote ± SEM.

## Results

### Initial evaluation of standard anticancer drugs

**Fig. 1:**
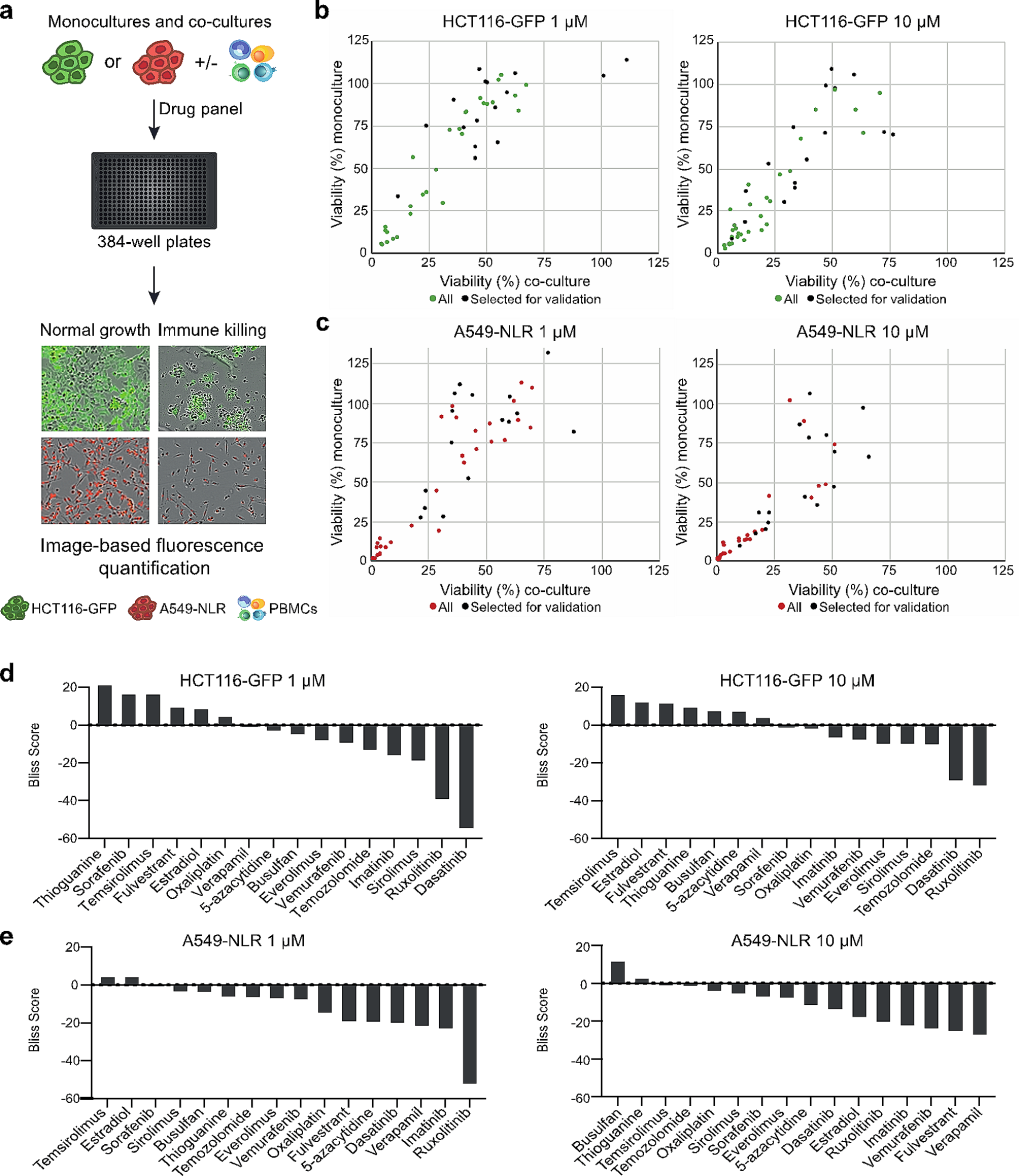
Initial evaluation of standard anticancer drugs. (**a**) The 384-well plate-based tumor-immune model system with mono- and co-cultures analyzed using an image-based readout. (**b**-**c**) The viability (expressed as % of control) in HCT116-GFP (**b**) and A549-NLR (**c**) monocultures and cancer-immune co-cultures after treatment with the drug panel at 1 µM and 10 µM for 72 h (data shown as means from three technical replicates). (**d**-**e**) Bliss scores calculated at 1 µM and 10 µM for the 16 drugs that were selected for validation experiments

To enable time and cost-effective evaluation of a broad panel of drugs in an immuno-oncology setting, we utilized a miniaturized in vitro tumor-immune model. The colon cancer cell line HCT116-GFP and the lung cancer cell line A549-NLR were grown as monocultures and co-cultured with anti-CD3/IL-2 activated PBMCs in a 384-well plate format. Monocultures and co-cultures were treated with a drug panel comprising 46 standard anticancer drugs and the viability of the cancer cells was indirectly measured by image-based quantification of fluorescence intensity (Fig. 1a). The drug panel was screened at 1 µM and 10 µM (Fig. 1b-c) and a Bliss score was calculated for each drug (Supplementary Data 1). Based on this synergy calculation, 16 drugs with Bliss scores ranging from the highest to the lowest were selected for validation experiments (Fig. 1d-e, Supplementary Data 1).

### Quality assessment of the assay

**Fig. 2:**
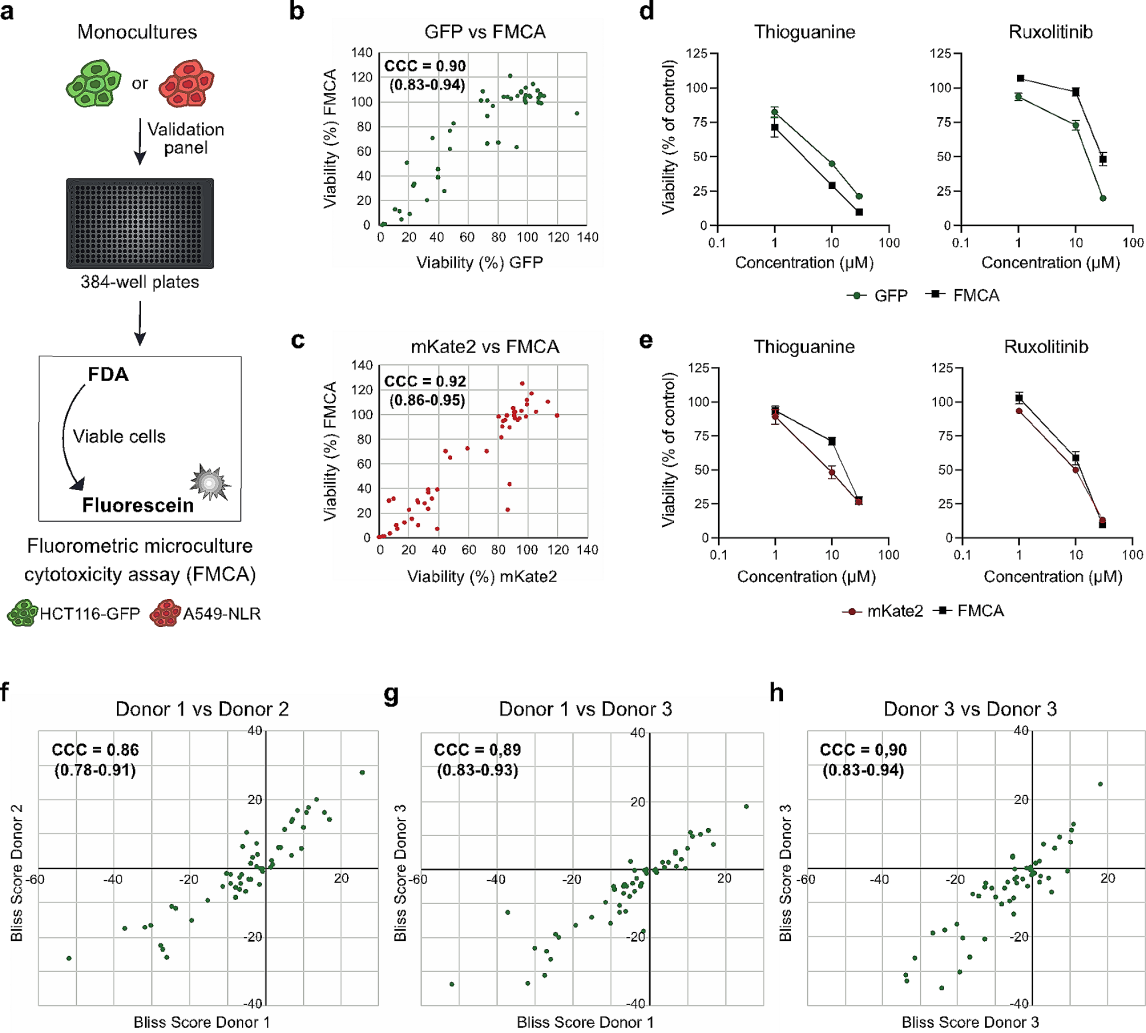
Quality assessment of the assay. (**a**) The 384-well plate-based model system with cancer cell monocultures analyzed using FMCA. (**b**-**c**) Viability of HCT116-GFP (**b**) and A549-NLR (**c**) cells, cultured as monocultures and treated with the validation drug panel at 1, 10, and 30 µM for 72 h, measured by FMCA and by image-based quantification of fluorescence. Correlation between the two assays determined by calculating Lin’s Concordance Correlation Coefficient (CCC). One representative experiment shown with data presented as means from three technical replicates. (**d**-**e**) Dose-response curves for the example drugs thioguanine and ruxolitinib. Viability measured in HCT116-GFP (**d**) and A549-NLR (**e**) with dual readouts after 72 h treatment. Data shown as mean ± SEM from three independent experiments. (**f**-**h**) Correlation between Bliss scores obtained with immune cells from three different donors (**f**-**g**) and between independent experiments performed with the same donor (**h**) determined by calculating CCC. Bliss Scores were calculated for HCT116-GFP after treatment with the validation drug panel at 1, 10, and 30 µM for 72 h

To verify the validity of using fluorescence intensity of constitutively expressed GFP or mKate2 as an indirect measure of cancer cell viability, dual readouts were used during initial validation experiments. HCT116-GFP and A549-NLR cells grown as monocultures were treated with the 16 selected drugs at 1, 10, and 30 µM and cancer cell viability was measured using FMCA [8] (Fig. 2a) in parallel with the image-based readout (Fig. 1a). Lin’s Concordance Correlation Coefficient (CCC) [10], a method commonly used to compare measurements of the same variable obtained with different assays, was used to assess the correlation between the viability measures. Three independent experiments were performed with CCC = 0.90 (0.83–0.94) (Fig. 2b), 0.88 (0.80–0.93), and 0.89 (0.82–0.94) (Supplementary Fig. S1a) for HCT116-GFP and CCC = 0.92 (0.86–0.95) (Fig. 2c), 0.92 (0.86–0.95), and 0.91 (0.86–0.95) (Supplementary Fig. S1b) for A549-NLR, demonstrating a clear correlation. As demonstrated also by the example drugs thioguanine and ruxolitinib, the two readouts generated equivalent results in both HCT116-GFP (Fig. 2d) and A549-NLR (Fig. 2e) cells. Subsequent experiments were performed using only the image-based readout. Next, both cell lines were grown as monocultures and as co-cultures with activated PBMCs from three different donors. Mono- and co-cultures were treated with the validation drug panel at 1, 10, and 30 µM. For each donor, a Bliss score was calculated for each drug and it was investigated whether the use of immune cells from different donors affected the reproducibility of the results. Concordance correlation analysis was performed and substantial correlations were observed both between data obtained from the different donors (Fig. 2f-g) and from independent experiments performed with the same donor (Fig. 2h) for HCT116-GFP and for A549-NLR (Supplementary Fig. S1c).

### Evaluation of standard anticancer drugs

**Fig. 3:**
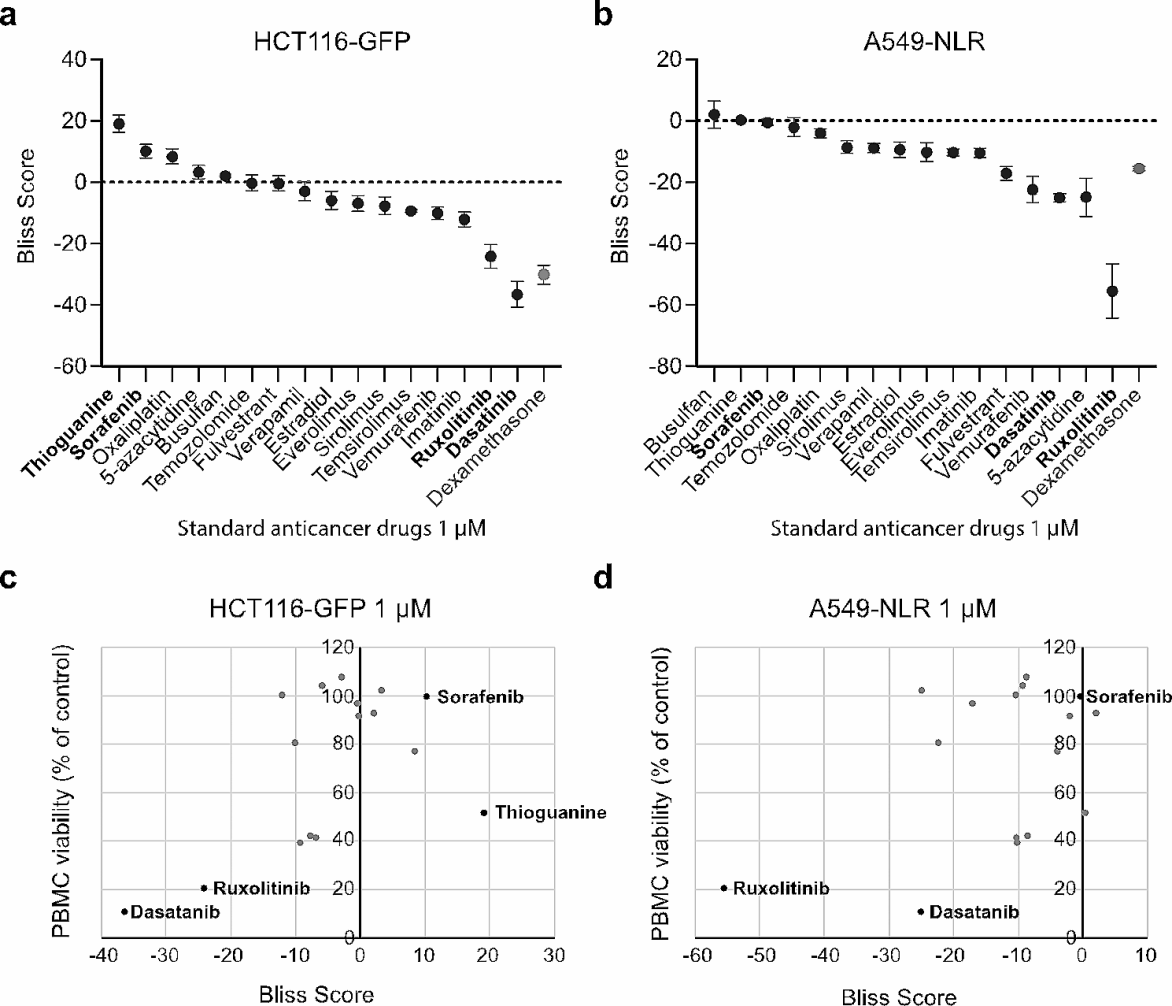
Evaluation of standard anticancer agents. (**a**-**b**) Bliss scores calculated after treatment with the validation drug panel and dexamethasone at 1µM for 72 h in HCT116-GFP (**a**) and A549-NLR (**b**). Data shown as mean ± SEM from three independent experiments. (**c**-**d**) Bliss scores (x-axis) plotted against the viability in PBMC monocultures (y-axis) after treatment with the validation drug panel at 1µM for 72 h in HCT116-GFP (**c**) and A549-NLR (**d**). Data shown as means from three independent experiments

Validation experiments were performed with the 16 selected anticancer drugs and dexamethasone, an immunosuppressive corticosteroid included as a positive control for antagonism. Results similar to those obtained during the initial drug evaluation were observed, with the greatest induction of synergy and antagonism occurring at 1 µM (Fig. 3a-b, Supplementary Data 2). The TKIs ruxolitinib and dasatinib generated the lowest Bliss scores (Fig. 3a-b). Overall, the correlation between Bliss scores and viability measures in PBMC monocultures after 72 h of drug treatment was weak (Fig. 3c-d). However, in the case of ruxolitinib and dasatinib, the observed antagonism with activated PBMCs can likely be explained by direct toxicity toward immune cells. At 1 µM, treatment with ruxolitinib or dasatinib had little to no direct effect on cancer cell viability while the viability of PBMCs was reduced to 20% and 10%, respectively (Fig. 3c-d). In contrast to other TKIs, the multi-kinase inhibitor sorafenib did not reduce the viability of PBMC at 1µM (Fig. 3c-d) and was shown to synergize with activated PBMCs at this concentration (Fig. 3a). However, toxicity was also observed for sorafenib at higher drug concentrations; the viability of the immune cells was, on average, reduced to 25% and 1% at 10 µM and 30 µM, respectively (Supplementary Data 2). In the HCT116-GFP model, the only anticancer agent that generated a higher Bliss score than sorafenib at 1 µM was thioguanine (Fig. 3a) and, although the viability of PBMCs alone was reduced to 35% at 10 µM, the Bliss score for thioguanine remained positive also at the higher drug concentration (Supplementary Data 2).

### Evaluation of concomitant drugs

**Fig. 4:**
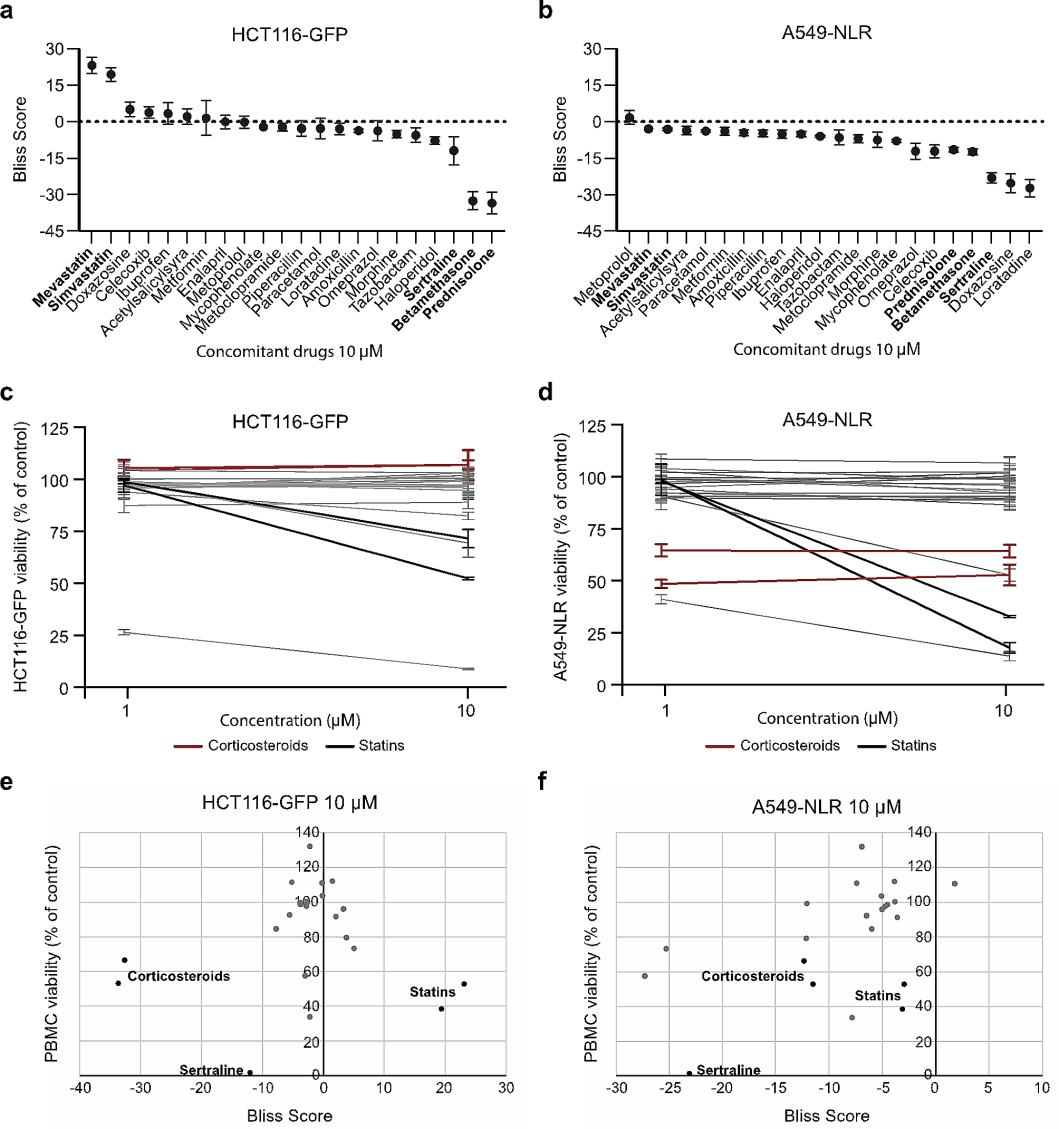
Evaluation of concomitant drugs. (**a**-**b**) Bliss scores calculated after treatment with concomitant drugs at 10 µM for 72 h in HCT116-GFP (**a**) and A549-NLR (**b**). Data shown as mean ± SEM from three independent experiments. (**c**-**d**) Viability in HCT116-GFP (**c**) and A549-NLR (**d**) monocultures treated with concomitant drugs for 72 h. Data shown as mean ± SEM from three independent experiments. (**e**-**f**) Bliss scores (x-axis) plotted against the viability in PBMC monocultures (y-axis) after treatment with concomitant drugs at 10 µM for 72 h in HCT116-GFP (**e**) and A549-NLR (**f**). Data shown as means from three independent experiments

Next, a drug panel comprised of 22 commonly prescribed concomitant drugs was evaluated using the same approach. As expected, the immunosuppressive corticosteroids betamethasone and prednisolone had the greatest antagonistic effect in the HCT116-GFP model (Fig. 4a). These drugs also induced a negative Bliss score in A549-NLR cells, but not to the same degree (Fig. 4b). The discrepancy between the two cell lines can be explained by their respective sensitivity to corticosteroids; while these drugs have no direct effect on the growth of HCT116-GFP cells, they have a well-documented inhibitory effect on the growth of lung cancer cells [12–14]. In agreement with the literature, we observed no decrease in HCT116-GFP cell viability (Fig. 4c) while the viability of A549-NLR cells was reduced to 53% and 65% after 72 h of treatment with betamethasone and prednisolone, respectively (Fig. 4d). Apart from the corticosteroids, the antidepressant sertraline was the only non-cancer drug that induced a Bliss score below − 10 in both cancer models at 10 µM (Fig. 4a-b, Supplementary data 3), and it was also the drug with the highest toxicity against PBMCs at this concentration (Fig. 4e-f). Overall, as for the standard anticancer agents, no apparent correlation was observed between Bliss scores and viability measures in PBMC monocultures after 72 h of treatment with concomitant drugs (Fig. 4e-f). Finally, in HCT116-GFP cells, the statins mevastatin and simvastatin were uniquely shown to synergize with activated PBMC at all tested drug concentrations (Fig. 2a and c, Supplementary Data 3). This was not observed in A549-NLR. Again, the discrepancy between the two cell lines can be explained by the different degrees of direct effect exerted on the cancer cells. Most concomitant drugs did not have any direct effect on cancer cell viability (Fig. 4c-d). Mevastatin and simvastatin reduced the viability of both HCT116-GFP (Fig. 4c) and A549-NLR (Fig. 4d) cells grown in monocultures. However, a much greater reduction of viability was observed in A549-NLR monocultures, leaving a smaller window for potentiation of immune cell-induced cancer cell death.

## Discussion

There are numerous assays available for in vitro evaluation of cancer cell viability and selecting a reliable method is critical. Quantification of stably expressed GFP has previously been established as a robust assay; a direct relationship has been demonstrated between the loss of GFP fluorescence and cell death induced by a variety of apoptotic stimuli [15]. To verify the validity of using image-based quantification of GFP and mKate2 as indirect measures of viability, we initially performed viability measures using FMCA [8] in parallel. Applying CCC analysis to assess the correlation between the two assays confirmed the feasibility of using image-based quantification of fluorescence to measure cancer cell viability (Fig. 2b-e. Supplementary Fig. S1a-b). Furthermore, CCC analysis demonstrated that the reproducibility of the results was not affected by the use of immune cells from different donors (Fig. 2f-g, Supplementary Fig. S1c). Taken together, these data demonstrate that our miniaturized tumor-immune model provides a robust platform for in vitro drug evaluation.

Upon evaluation of a broad panel of standard anticancer drugs, TKIs were found to be among the top inducers of both antagonism and synergy. Treatment with ruxolitinib and dasatinib induced the lowest Bliss scores (Fig. 3a-b). Ruxolitinib is a specific inhibitor of the tyrosine kinases JAK1 and JAK2. The JAK-STAT signaling pathway plays a critical role in signal transduction in cells of the immune system and ruxolitinib is known to target various cellular components of both innate and adaptive immunity [16]. As for dasatinib, a TKI used for the treatment of myeloid and lymphoblastic leukemias, effective inhibition of T-cell activation and proliferation have previously been reported [17]. The TKI sorafenib was, on the contrary, shown to synergize with activated PBMCs at 1 µM (Fig. 3a). At standard dosing, sorafenib reaches a maximum plasma concentration (Cmax) of around 20 µM in patients [18]. At this concentration, sorafenib has been shown to induce immunosuppression. For example, Iyver et al., demonstrated that treatment with high dose (10 µM) but not low dose (1 µM) sorafenib results in decreased T cell proliferation and an increased proportion of PD-1 expressing CD8 + T cells in vitro [19]. Furthermore, using an in vivo woodchuck model for hepatocellular carcinoma, they found that low-dose sorafenib treatment not only generated a significantly greater delay in tumor growth than high-dose treatment but also a significant increase in CD3 + T cells in the tumors. Along the same line, according to a study by Cabrera et al., pharmacologic doses of sorafenib decreases the activation of effector T cells, whereas sub-pharmacologic doses result in a selective increase in the activation of effector T cells and suppression of regulatory T cells [20]. In agreement with the literature, we observed synergy between activated immune cells and sorafenib treatment at 1 µM but not at 10 or 30 µM. Furthermore, at the higher concentrations, the viability of activated immune cells in monoculture was reduced to only 25% and 1%, respectively (Supplementary data 2). These data, generated by us and others, highlights the importance of balancing cytotoxicity toward tumor cells versus immune cells when designing treatment regimens.

When evaluating commonly used concomitant drugs, the immunosuppressive corticosteroids betamethasone and prednisolone emerged as the most antagonistic substances (Fig. 4), demonstrating the ability of the model system to capture known immunological features. Treatment with sertraline also induced antagonism in both cancer models at 10 µM and it was the non-cancer drug with the by far highest toxicity against PBMCs (Fig. 4). Previous studies performed in murine models suggest that sertraline, along with other selective serotonin reuptake inhibitors (SSRIs), may exert immunosuppressive effects [21, 22]. Notably, the sertraline-concentrations used in the present study were substantially higher than those reachable in patients. Further investigations into the potential anti-inflammatory effects of sertraline in a human model system at more clinically relevant concentrations should be performed. Most concomitant drugs had little to no direct effect on the viability of the cancer cells, however, the statins mevastatin and simvastatin reduced the viability of both HCT116-GFP and A549-NLR cells in monoculture (Fig. 4c-d). A considerable amount of data from both preclinical and clinical studies has demonstrated that statins, i.e., widely prescribed cholesterol-lowering drugs, possess tumor-suppressing properties [23]. In HCT116-GFP cells, mevastatin and simvastatin were also uniquely shown to synergize with activated PBMC at all tested drug concentrations (Fig. 4a and c, Supplementary Data 3). However, this was not observed in the A549-NLR model. In monocultures, 10 µM simvastatin reduced the viability of HCT116-GFP and A549-NLR cells to 52% and 19%, respectively (Fig. 4c-d). The presence of activated PBMCs during simvastatin treatment further reduced the cancer cell viability to 16% in HCT116-GFP and to 11% in A549-NLR (data not shown). Thus, the discrepancy between the two cell lines can likely be explained by their respective sensitivities to statin treatment as a much smaller window for potentiation of immune cell-induced cancer cell death was present in the A549-NLR model.

The observation that statins synergize with activated PBMCs is in line with previous findings in our group; aiming to identify immunomodulatory small molecule drugs with potential applications in cancer treatment, we recently performed a drug repurposing screen [5]. Among the 1,280 FDA-approved drugs that were screened, mevastatin stood out as one of few drugs with the ability to enhance immune cell-induced cancer cell death. Further investigation demonstrated that this feature is shared by other lipophilic statins such as simvastatin and pitavastatin. In the present study, the highest Bliss scores were obtained at 10 µM for both statins, which is not an achievable concentration in patients [24]. However, synergy between statins and activated immune cells was observed already at 1 µM (Supplementary data 3), a concentration close to those attainable in patients. Furthermore, increasing data supports the notion that statins may exert pro-inflammatory effects also in patients; several recent studies suggest that statin treatment is associated with improved clinical outcomes for cancer patients receiving therapy with immune checkpoint inhibitors [25–28].

The evaluation of large panels of drugs in an immuno-oncology setting in vitro is a challenging task. In our in vitro model, we co-cultured human colon and lung cancer cell lines with PBMCs sourced from different donors, as has been performed previously by us and others. It is highly reproducible and, compared to using primary autologous cells, a relatively straightforward assay. Nonetheless, as the model is crude, it is prudent to speculate on what aspects of the anti-tumor immune response it can recapitulate. The PBMC provides the model with agents of adaptive (B and T lymphocytes) as well as innate (monocytes, NK, dendritic cells) immunity but omits granulocytes [29]. The model also omits other cells that can modulate the immune response [30], including but not limited to myeloid-derived suppressor cells [31] and cancer-associated fibroblasts [32]. Hence, the model reflects a simplified version of immune interactions in the TME, focusing on elements provided by PBMCs and their short-term interactions with each other and the target cells. Furthermore, the adaptive immune response in the model is not tumor antigen-specific. In the canonical model of immune activation, the response is primed by dendritic cells presenting antigens in secondary lymph organs, whereas in this model peripheral T-cells are activated and proliferated independent of antigen by the addition of ⍺CD3 and IL-2. Also, the adaptive immune response of the model is alloreactive as the PBMC donors have not been matched with the cell lines. It has been estimated that about 10% of T-cells are alloreactive, orders of magnitude more than react to specific peptide antigens [33]. It follows that the model is likely ill-suited for the detection of immunomodulation arising due to shifts in which peptides are presented on MHC. However, the ⍺CD3 used, which binds CD3𝛆, as well as T-cell allorecognition, although not specific to the peptide presented, induce T cell receptor (TCR) signaling [34, 35]. Thus, the model should be sensitive to intracellular interference with TCR signaling which is supported by the antagonism observed for glucocorticoids, reflecting a step in the immune response which is necessary but not sufficient for initiation of T-cell mediated cell killing. Given the relatively high frequency of reactive cells due to alloreactivity, we suggest that the model used herein mostly resembles an ongoing immune reaction in a “hot” tumor on a short time scale and that synergy or antagonism indicates a compound-induced reshaping of that immune response. Although beyond the scope of this paper, the underlying mechanism could then be further deconvolved by e.g., flow cytometry on treated cultures to identify changes in the cell type composition, selective depletion of immune subpopulations, or cytokine and gene expression profiling to provide clues as to which if any in vivo settings the phenomenon translates to.

## Conclusions

In summary, our miniaturized tumor-immune model, while not exhaustive, has demonstrated ability to capture previously described immunological effects of anticancer agents, such as the dose-dependent immunomodulatory feature of sorafenib. Additionally, it can be readily adapted to include a wide range of cancer cell types and/ or specific subpopulations of immune cells. Thus, we believe that it offers a valuable starting point for identifying potential drug-cell interactions that warrant further, more detailed investigation.

### Electronic supplementary material

Below is the link to the electronic supplementary material.

Supplementary Material 1

## Data Availability

All data and materials are available within the article and its supplementary data files.
