# Supplementary material for: Immuno-oncological effects of standard anticancer agents and commonly used concomitant drugs: an in vitro assessment
Source: BMC Pharmacol Toxicol. 2024 Mar 5;25:25. doi: 10.1186/s40360-024-00746-6 (PMC10913607; doi:10.1186/s40360-024-00746-6)
Supplement: Supplementary file 1 — Supplementary Material 1 [file 40360_2024_746_MOESM1_ESM.pdf]

Supplementary Information to:

Immuno-oncological effects of standard anticancer agents and commonly used concomitant drugs: an in vitro assessment.

Tove Selvin[1]*, Malin Berglund[1], Lena Lenhammar[1], Magnus Lindskog[2], Malin Jarvius[3], Rolf Larsson[1], Peter Nygren[2], Mårten Fryknäs[1] and Claes R Andersson[1]*

[1] Department of Medical Sciences, Division of Cancer Pharmacology and Computational Medicine, Uppsala University, SE-75185 Uppsala, Sweden.
[2] Department of Immunology, Genetics and Pathology, Uppsala University, SE-75185 Uppsala, Sweden.
[3] Department of Pharmaceutical Biosciences and Science for Life Laboratory, Uppsala University, Box 591, SE-751 24 Uppsala, Sweden

*Correspondence to Tove Selvin (tove.selvin@medsci.uu.se) and Claes R Andersson (claes.andersson@medsci.uu.se)

Journal: BMC Pharmacology and Toxicology

Supplementary Figures

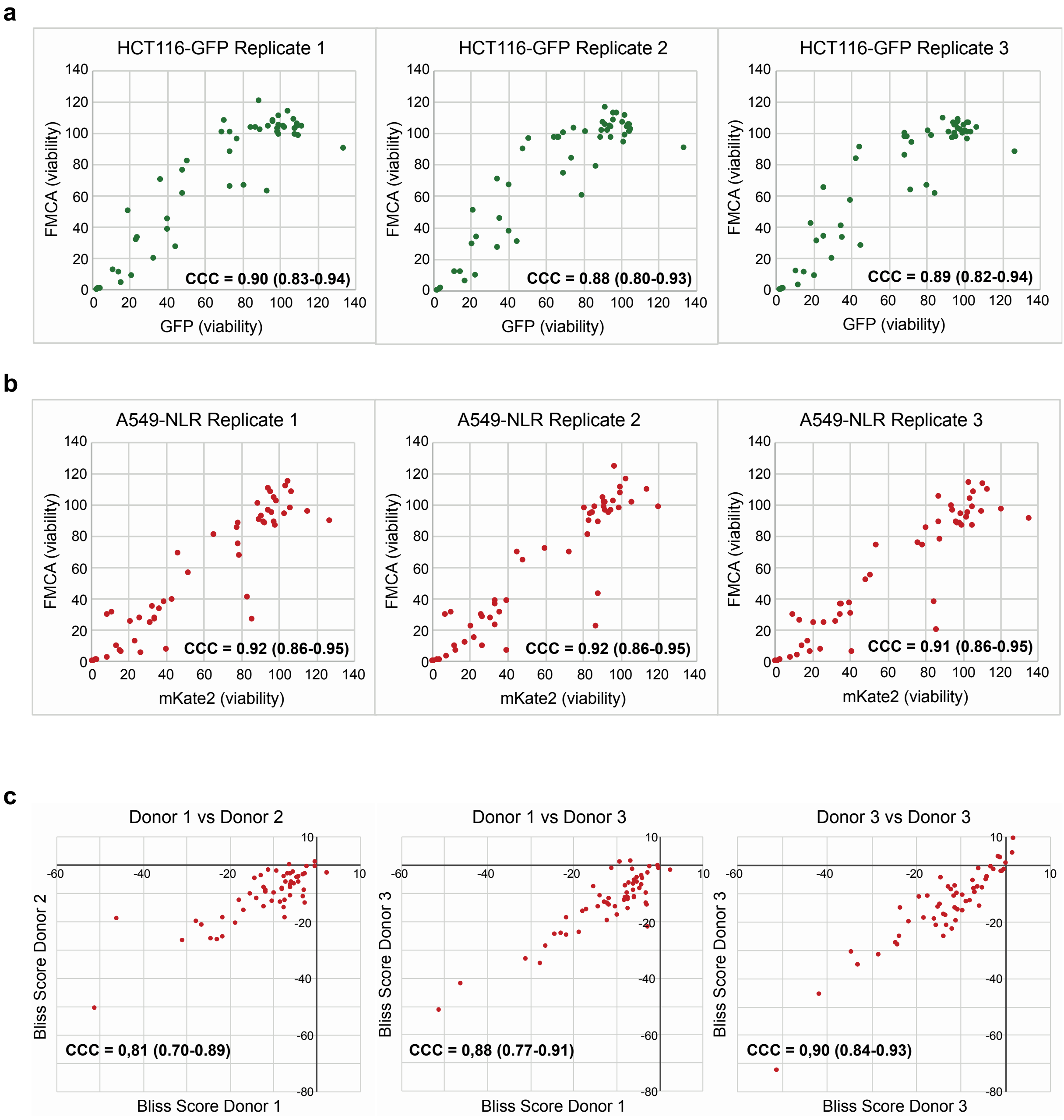

Fig. S1

Viability of HCT116-GFP (a) and A549-NLR (b) cells, cultured as monocultures and treated with the validation drug panel at 1, 10, and 30 µM for 72 h, measured by FMCA and by image-based quantification of fluorescence. Correlation between the two assays determined by calculating Lin's Concordance Correlation Coefficient (CCC). (c) Correlation between Bliss Scores obtained with immune cells from three different donors and between independent experiments performed with the same donor determined by calculating CCC. Bliss Scores were calculated for A549-NLR after treatment with the validation drug panel at 1, 10, and 30 µM for 72 h.

Supplementary Data

Supplementary Data 1

Calculated Bliss Scores from the initial drug panel evaluation

SIt = Viability induced by drug **SIe = Viability induced by drug + PBMC** **SIi = Viability induced by PBMC**

☐ Marks drugs selected for validation

Std treatments	Drug class	Conc.	HCT116-GFP monoculture			SIt Avg	HCT116-GFP co-culture			SIe Avg	SIi	Bliss Score SIt*SIi - SIe
Thioguanine	Antimetabolite	1uM	72,8	76,1	76,8	**75,2**	24,9	22,7	24,4	**24,0**	**58,6**	20
Sorafenib	Kinase inhibitor	1uM	106,2	83,3	81,8	**90,5**	31,5	37,1	39,2	**35,9**	**58,3**	17
Temsirolimus	mTOR-inhibitor	1uM	104,7	116,5	104,2	**108,5**	49,6	40,6	50,5	**46,9**	**58,6**	17
Fulvestrant	Hormone treatment	1uM	105,8	103,2	95,2	**101,4**	56,4	47,6	44,8	**49,6**	**58,6**	10
Estradiol	Hormone treatment	1uM	104,8	99,5	98,4	**100,9**	45,6	54,6	50,5	**50,2**	**58,6**	9
Oxaliplatin	Alkylating agent	1uM	33,5	32,0	34,6	**33,3**	11,0	11,3	12,2	**11,5**	**48,6**	5
Verapamil	Calcium-channel blc	1uM	112,1	97,1	109,0	**106,1**	63,8	60,4	63,6	**62,6**	**58,6**	0
Busulfan	Alkylating agent	1uM	91,7	97,8	94,4	**94,6**	63,4	56,7	57,2	**59,1**	**58,6**	-4
5-azacytidine	Antimetabolite	1uM	73,7	77,1	71,4	**74,1**	42,4	31,1	46,4	**40,0**	**46,4**	-6
Everolimus	mTOR-inhibitor	1uM	61,6	67,8	58,8	**62,7**	47,7	43,6	44,3	**45,2**	**58,6**	-8
Imatinib	Kinase inhibitor	1uM	71,0	81,1	81,8	**77,9**	44,0	54,0	39,8	**45,9**	**46,4**	-10
Vemurafenib	Kinase inhibitor	1uM	88,6	90,9	78,8	**86,1**	50,9	57,1	53,7	**53,9**	**46,4**	-14
Temozolomide	Alkylating agent	1uM	68,5	56,9	71,2	**65,5**	60,3	44,4	60,4	**55,0**	**58,6**	-17
Sirolimus	mTOR-inhibitor	1uM	55,9	56,1	55,8	**55,9**	46,4	42,2	47,0	**45,2**	**46,4**	-19
Ruxolitinib	Kinase inhibitor	1uM	108,5	105,8	99,4	**104,6**	102,0	94,6	105,9	**100,8**	**58,3**	-40
Dasatinib	Kinase inhibitor	1uM	113,6	114,0	114,8	**114,1**	106,5	124,8	101,5	**110,9**	**48,7**	-55
Palbociclib	Kinase inhibitor	1uM	101,5	99,9	100,7	**100,7**	49,0	51,0	50,5	**50,2**	**58,3**	8
Alectinib	Kinase inhibitor	1uM	60,5	53,7	56,1	**56,8**	17,5	19,3	17,5	**18,1**	**40,9**	5
Venetoclax	BCL-2 inhibitor	1uM	96,6	101,5	109,0	**102,3**	55,5	54,0	56,1	**55,2**	**58,3**	4
Vorinostat	HDAC inhibitor	1uM	72,8	73,9	72,5	**73,1**	33,5	39,7	41,8	**38,3**	**58,3**	4
Irinotecan	Topoisomerase inhil	1uM	69,3	72,9	76,5	**72,9**	35,2	31,3	35,3	**33,9**	**48,6**	2
Paclitaxel	Microtubule inhibit	1uM	13,7	17,8	14,4	**15,3**	5,9	6,4	6,0	**6,1**	**46,4**	1
Vinorelbine	Microtubule inhibit	1uM	14,2	11,6	14,7	**13,5**	5,2	7,4	5,7	**6,1**	**46,4**	0
Fluorouracil	Antimetabolite	1uM	85,3	82,8	80,4	**82,9**	55,4	29,4	37,4	**40,7**	**48,6**	0
Vincristine	Microtubule inhibit	1uM	12,8	13,0	11,0	**12,3**	6,8	6,4	6,9	**6,7**	**46,4**	-1
Doxorubicin	Anthracycline	1uM	5,4	5,7	5,2	**5,4**	4,6	4,8	3,7	**4,4**	**50,9**	-2
Tamoxifen	Hormone treatment	1uM	87,8	94,7	95,8	**92,7**	62,8	61,5	63,7	**62,7**	**65,5**	-2
Epirubicin	Anthracycline	1uM	5,4	4,8	5,4	**5,2**	4,9	4,7	4,4	**4,7**	**50,9**	-2
Bendamustine	Alkylating agent	1uM	96,6	105,3	95,7	**99,2**	69,1	71,3	61,0	**67,1**	**65,5**	-2
Melphalan	Alkylating agent	1uM	78,6	76,6	95,4	**83,5**	39,2	42,9	41,7	**41,2**	**46,4**	-2
Gemcitabine	Antimetabolite	1uM	29,1	27,3	27,1	**27,8**	16,3	17,0	17,7	**17,0**	**50,9**	-3
Octreotide	Hormone treatment	1uM	84,8	101,9	87,9	**91,5**	40,0	48,6	53,8	**47,5**	**48,7**	-3
Gefitinib	Kinase inhibitor	1uM	106,2	105,3	104,7	**105,4**	58,3	52,4	59,1	**56,6**	**50,9**	-3
Amsacrine	Acridine	1uM	38,3	34,8	34,3	**35,8**	22,8	26,3	22,8	**24,0**	**58,3**	-3
Etoposide	Topoisomerase inhil	1uM	65,6	72,4	73,1	**70,4**	42,4	32,0	43,6	**39,3**	**50,9**	-4
Daunorubicin	Anthracycline	1uM	5,9	6,7	6,4	**6,3**	7,1	6,7	6,9	**6,9**	**50,9**	-4
Carboplatin	Alkylating agent	1uM	81,5	87,0	97,3	**88,6**	51,4	48,2	47,0	**48,9**	**50,9**	-4
Regorafenib	Kinase inhibitor	1uM	44,8	51,3	51,6	**49,2**	24,3	27,7	32,3	**28,1**	**48,6**	-4
Dactinomycin	Anthracycline	1uM	8,5	9,1	8,1	**8,6**	9,4	9,5	8,9	**9,3**	**58,6**	-4

Mitoxantrone	Anthracycline	1uM	22,6	21,7	25,1	**23,1**	17,6	16,1	16,7	**16,8**	**48,7**	-6
Mitomycin	Anthracycline	1uM	34,2	34,1	35,1	**34,5**	23,2	22,6	21,2	**22,4**	**48,7**	-6
Idarubicin	Anthracycline	1uM	9,5	8,6	10,6	**9,5**	10,0	10,7	13,0	**11,2**	**50,9**	-6
Olaparib	PARP inhibitor	1uM	88,4	93,0	82,7	**88,0**	49,6	56,8	44,3	**50,2**	**48,7**	-7
Erlotinib	Kinase inhibitor	1uM	88,5	87,7	90,2	**88,8**	53,9	50,7	53,5	**52,7**	**50,9**	-8
Ponatinib	Kinase inhibitor	1uM	27,5	29,9	31,2	**29,5**	28,9	33,6	31,0	**31,1**	**58,3**	-14
Sunitinib	Kinase inhibitor	1uM	82,7	81,8	87,2	**83,9**	68,7	60,5	62,8	**64,0**	**58,3**	-15

Std treatments	Drug class	Conc.	HCT116-GFP monoculture			Slt Avg	HCT116-GFP co-culture			Sle Avg	Sli	Slt*Sli - Sie
Temsirolimus	mTOR-inhibitor	10uM	109,7	115,1	102,0	**108,9**	45,8	53,1	50,0	**49,6**	**60,5**	16
Estradiol	Hormone treatment	10uM	106,1	92,8	98,1	**99,0**	45,4	43,5	53,1	**47,3**	**60,5**	13
Fulvestrant	Hormone treatment	10uM	69,6	74,0	79,7	**74,4**	31,1	34,7	33,5	**33,1**	**60,5**	12
Thioguanine	Antimetabolite	10uM	35,5	33,7	41,1	**36,8**	11,7	13,2	12,7	**12,5**	**60,5**	10
Busulfan	Alkylating agent	10uM	92,7	107,3	93,1	**97,7**	54,1	45,5	54,0	**51,2**	**60,5**	8
5-azacytidine	Antimetabolite	10uM	51,1	53,6	54,8	**53,2**	20,1	24,2	21,8	**22,1**	**55,7**	8
Verapamil	Calcium-channel blc	10uM	115,5	102,3	99,1	**105,6**	60,4	59,1	59,2	**59,6**	**60,5**	4
Sorafenib	Kinase inhibitor	10uM	9,9	8,1	9,1	**9,0**	6,7	5,9	6,7	**6,4**	**62,9**	-1
Oxaliplatin	Alkylating agent	10uM	20,1	17,3	17,9	**18,5**	11,2	13,1	12,1	**12,1**	**52,8**	-2
Imatinib	Kinase inhibitor	10uM	69,4	66,6	77,7	**71,2**	45,9	49,9	44,2	**46,7**	**55,7**	-7
Vemurafenib	Kinase inhibitor	10uM	55,7	58,3	51,8	**55,3**	36,4	43,8	36,7	**39,0**	**55,7**	-8
Everolimus	mTOR-inhibitor	10uM	38,3	35,5	42,2	**38,6**	40,5	29,4	31,8	**33,9**	**60,5**	-11
Sirolimus	mTOR-inhibitor	10uM	41,7	44,5	38,2	**41,5**	32,6	30,3	38,3	**33,8**	**55,7**	-11
Temozolomide	Alkylating agent	10uM	26,7	34,6	30,4	**30,6**	31,3	29,4	27,0	**29,2**	**60,5**	-11
Dasatinib	Kinase inhibitor	10uM	69,6	68,4	76,5	**71,5**	71,8	78,1	67,3	**72,4**	**59,4**	-30
Ruxolitinib	Kinase inhibitor	10uM	73,0	66,9	70,3	**70,1**	77,6	78,4	74,0	**76,7**	**62,9**	-33
Paclitaxel	Microtubule inhibit	10uM	25,4	25,1	27,3	**25,9**	5,8	5,7	5,3	**5,6**	**55,7**	9
Fluorouracil	Antimetabolite	10uM	37,8	43,0	40,9	**40,6**	13,9	13,3	13,4	**13,5**	**52,8**	8
Carboplatin	Alkylating agent	10uM	92,2	79,8	82,1	**84,7**	44,5	39,4	44,4	**42,8**	**58,8**	7
Octreotide	Hormone treatment	10uM	96,8	95,7	97,0	**96,5**	51,4	54,2	48,4	**51,3**	**59,4**	6
Olaparib	PARP inhibitor	10uM	71,0	66,8	65,7	**67,8**	32,0	40,6	36,2	**36,3**	**59,4**	4
Irinotecan	Topoisomerase inhil	10uM	29,3	27,7	29,4	**28,8**	14,5	15,0	12,8	**14,1**	**52,8**	1
Vincristine	Microtubule inhibit	10uM	12,5	13,9	14,1	**13,5**	6,9	6,8	6,1	**6,6**	**55,7**	1
Vinorelbine	Microtubule inhibit	10uM	15,2	12,6	16,0	**14,6**	8,9	7,7	7,6	**8,1**	**55,7**	0
Tamoxifen	Hormone treatment	10uM	76,0	94,3	84,4	**84,9**	65,5	55,8	59,1	**60,1**	**70,8**	0
Mitomycin	Anthracycline	10uM	9,9	10,2	9,5	**9,9**	6,5	5,7	6,3	**6,2**	**59,4**	0
Mitoxantrone	Anthracycline	10uM	4,1	4,7	5,2	**4,7**	3,4	2,5	3,5	**3,1**	**59,4**	0
Alectinib	Kinase inhibitor	10uM	13,5	15,1	21,2	**16,6**	4,3	11,2	6,8	**7,4**	**40,7**	-1
Venetoclax	BCL-2 inhibitor	10uM	33,2	29,0	36,4	**32,8**	22,0	22,0	20,8	**21,6**	**62,9**	-1
Melphalan	Alkylating agent	10uM	49,8	47,2	42,3	**46,4**	24,6	26,8	30,3	**27,2**	**55,7**	-1
Ponatinib	Kinase inhibitor	10uM	3,2	2,6	3,1	**2,9**	3,1	3,0	3,6	**3,2**	**62,9**	-1
Amsacrine	Acridine	10uM	5,5	6,6	5,0	**5,7**	4,2	6,1	5,2	**5,2**	**62,9**	-2
Regorafenib	Kinase inhibitor	10uM	5,2	6,0	5,1	**5,4**	4,9	5,1	5,3	**5,1**	**52,8**	-2
Doxorubicin	Anthracycline	10uM	11,6	11,4	11,9	**11,6**	8,5	10,2	9,4	**9,4**	**58,8**	-3
Dactinomycin	Anthracycline	10uM	5,9	5,7	6,2	**5,9**	5,6	6,9	6,4	**6,3**	**60,5**	-3
Vorinostat	HDAC inhibitor	10uM	8,3	11,2	9,5	**9,7**	8,8	9,1	8,8	**8,9**	**62,9**	-3
Erlotinib	Kinase inhibitor	10uM	53,7	44,9	47,6	**48,7**	31,4	33,2	30,6	**31,7**	**58,8**	-3
Epirubicin	Anthracycline	10uM	10,6	12,3	10,9	**11,3**	10,1	9,3	11,7	**10,4**	**58,8**	-4
Bendamustine	Alkylating agent	10uM	94,7	94,3	95,6	**94,8**	69,3	73,1	70,3	**70,9**	**70,8**	-4

Etoposide	Topoisomerase inhil	10uM	29,4	35,3	28,6	**31,1**	23,9	24,7	20,0	**22,9**	**58,8**	-5
Gemcitabine	Antimetabolite	10uM	22,7	24,1	20,1	**22,3**	20,9	18,5	18,0	**19,1**	**58,8**	-6
Daunorubicin	Anthracycline	10uM	11,8	13,6	12,1	**12,5**	13,3	13,6	13,4	**13,4**	**58,8**	-6
Sunitinib	Kinase inhibitor	10uM	7,5	8,3	6,9	**7,6**	11,1	11,7	12,6	**11,8**	**62,9**	-7
Palbociclib	Kinase inhibitor	10uM	18,5	16,6	16,7	**17,2**	21,4	18,2	24,6	**21,4**	**62,9**	-11
Idarubicin	Anthracycline	10uM	12,4	15,6	13,1	**13,7**	20,0	17,5	20,0	**19,2**	**58,8**	-11
Gefitinib	Kinase inhibitor	10uM	76,8	63,5	73,4	**71,3**	68,0	62,5	60,2	**63,6**	**58,8**	-22

Std treatments	Drug class	Conc.	A549-NLR monoculture			Slt Avg	A549-NLR co-culture			Sle Avg	Sli	Slt*Sli - Sie
Temsirolimus	mTOR-inhibitor	1uM	108,2	94,9	114,3	**105,8**	36,7	38,5	34,5	**36,6**	**39,1**	5
Estradiol	Hormone treatment	1uM	110,6	111,2	112,7	**111,5**	35,5	38,3	42,8	**38,9**	**39,1**	5
Sorafenib	Kinase inhibitor	1uM	74,8	111,5	97,5	**94,6**	40,6	36,1	29,9	**35,5**	**37,9**	0
Sirolimus	mTOR-inhibitor	1uM	39,6	31,5	28,0	**33,0**	21,2	27,6	21,6	**23,5**	**59,1**	-4
Busulfan	Alkylating agent	1uM	95,9	112,2	106,5	**104,9**	39,1	53,5	39,6	**44,1**	**37,9**	-4
Thioguanine	Antimetabolite	1uM	70,3	57,4	96,8	**74,8**	36,6	33,3	35,5	**35,1**	**37,9**	-7
Temozolomide	Alkylating agent	1uM	44,5	49,3	38,9	**44,2**	26,0	21,7	23,8	**23,8**	**37,9**	-7
Everolimus	mTOR-inhibitor	1uM	20,9	31,6	28,8	**27,1**	24,0	21,3	20,3	**21,8**	**52,2**	-8
Vemurafenib	Kinase inhibitor	1uM	66,3	92,8	103,9	**87,7**	66,0	70,9	43,3	**60,1**	**59,1**	-8
Oxaliplatin	Alkylating agent	1uM	35,1	50,2	70,4	**51,9**	41,2	44,3	41,9	**42,5**	**52,2**	-15
Fulvestrant	Hormone treatment	1uM	112,3	92,1	107,3	**103,9**	46,5	57,4	77,1	**60,3**	**39,1**	-20
5-azacytidine	Antimetabolite	1uM	132,5	131,7	131,2	**131,8**	78,2	75,4	76,9	**76,8**	**43,1**	-20
Dasatinib	Kinase inhibitor	1uM	31,4	27,4	23,5	**27,4**	33,1	33,5	27,7	**31,4**	**39,1**	-21
Verapamil	Calcium-channel blc	1uM	101,8	78,1	86,1	**88,7**	68,7	49,4	52,9	**57,0**	**39,1**	-22
Imatinib	Kinase inhibitor	1uM	102,2	88,1	88,6	**93,0**	61,5	66,8	62,7	**63,7**	**43,1**	-24
Ruxolitinib	Kinase inhibitor	1uM	71,6	83,6	89,9	**81,7**	69,0	101,3	94,0	**88,1**	**43,1**	-53
Olaparib	PARP inhibitor	1uM	86,6	101,4	85,6	**91,2**	30,0	34,5	27,6	**30,7**	**39,1**	5
Octreotide	Hormone treatment	1uM	102,7	104,4	85,9	**97,7**	33,3	36,0	36,7	**35,3**	**39,1**	3
Paclitaxel	Microtubule inhibit	1uM	7,4	8,5	8,2	**8,1**	2,9	2,3	2,6	**2,6**	**59,1**	2
Vinorelbine	Microtubule inhibit	1uM	11,9	15,7	14,1	**13,9**	4,5	3,2	4,6	**4,1**	**43,1**	2
Vincristine	Microtubule inhibit	1uM	12,4	10,4	9,4	**10,7**	3,2	3,0	2,9	**3,1**	**43,1**	2
Mitomycin	Anthracycline	1uM	7,5	9,5	8,7	**8,6**	4,9	4,0	4,4	**4,4**	**50,9**	0
Idarubicin	Anthracycline	1uM	1,1	1,3	1,2	**1,2**	1,0	1,1	1,2	**1,1**	**59,1**	0
Daunorubicin	Anthracycline	1uM	1,2	1,1	1,3	**1,2**	1,0	1,5	1,4	**1,3**	**52,2**	-1
Epirubicin	Anthracycline	1uM	1,6	1,4	1,2	**1,4**	1,6	2,5	1,4	**1,8**	**52,2**	-1
Gemcitabine	Antimetabolite	1uM	4,5	4,4	4,6	**4,5**	3,9	4,4	4,0	**4,1**	**59,1**	-1
Bendamustine	Alkylating agent	1uM	70,1	102,3	99,5	**90,6**	42,4	39,0	29,9	**37,1**	**39,1**	-2
Dactinomycin	Anthracycline	1uM	3,7	2,8	3,3	**3,3**	2,9	2,7	4,0	**3,2**	**37,9**	-2
Doxorubicin	Anthracycline	1uM	3,7	4,3	4,2	**4,1**	4,2	3,7	4,5	**4,1**	**52,2**	-2
Mitoxantrone	Anthracycline	1uM	7,8	9,0	7,9	**8,2**	6,8	7,7	5,9	**6,8**	**50,9**	-3
Carboplatin	Alkylating agent	1uM	74,6	90,6	80,4	**81,9**	37,0	46,0	53,5	**45,5**	**52,2**	-3
Amsacrine	Acridine	1uM	12,1	11,5	10,9	**11,5**	8,2	7,9	9,9	**8,7**	**37,9**	-4
Melphalan	Alkylating agent	1uM	76,0	63,8	71,9	**70,6**	39,3	50,8	48,0	**46,0**	**59,1**	-4
Etoposide	Topoisomerase inhil	1uM	18,8	22,7	24,4	**21,9**	16,5	18,9	18,1	**17,8**	**59,1**	-5
Fluorouracil	Antimetabolite	1uM	103,1	83,7	74,2	**87,0**	55,8	44,9	54,4	**51,7**	**52,2**	-6
Irinotecan	Topoisomerase inhil	1uM	61,9	63,7	60,1	**61,9**	38,9	40,5	42,1	**40,5**	**52,2**	-8
Palbociclib	Kinase inhibitor	1uM	42,6	43,6	45,8	**44,0**	30,1	28,5	27,5	**28,7**	**43,1**	-10
Tamoxifen	Hormone treatment	1uM	107,0	104,8	91,5	**101,1**	60,8	71,9	53,1	**61,9**	**50,9**	-10

Erlotinib	Kinase inhibitor	1uM	80,7	84,1	63,5	**76,1**	62,4	53,0	59,1	**58,2**	**59,1**	-13
Regorafenib	Kinase inhibitor	1uM	72,4	79,9	72,7	**75,0**	54,9	64,7	37,8	**52,5**	**52,2**	-13
Vorinostat	HDAC inhibitor	1uM	72,7	65,6	60,9	**66,4**	45,4	34,9	38,6	**39,6**	**37,9**	-15
Alectinib	Kinase inhibitor	1uM	94,0	89,8	83,1	**89,0**	60,2	63,8	67,7	**63,9**	**50,9**	-19
Gefitinib	Kinase inhibitor	1uM	83,5	74,6	94,8	**84,3**	67,2	69,6	71,1	**69,3**	**59,1**	-20
Ponatinib	Kinase inhibitor	1uM	13,5	23,2	20,0	**18,9**	20,8	35,4	33,3	**29,8**	**43,1**	-22
Sunitinib	Kinase inhibitor	1uM	104,5	115,1	118,5	**112,7**	75,5	55,6	64,8	**65,3**	**37,9**	-23
Venetoclax	BCL-2 inhibitor	1uM	105,1	116,1	107,0	**109,4**	54,4	79,3	76,8	**70,2**	**37,9**	-29

**Std treatments**	**Drug class**	**Conc.**	**A549-NLR monoculture**			**Slt Avg**	**A549-NLR co-culture**			**Sle Avg**	**Sli**	**Slt*Sli - Sie**
Busulfan	Alkylating agent	10uM	71,8	98,7	89,8	**86,8**	30,5	42,5	36,0	**36,3**	**55,7**	12
Thioguanine	Antimetabolite	10uM	84,3	81,1	68,8	**78,1**	44,3	33,4	43,3	**40,3**	**55,7**	3
Temsirolimus	mTOR-inhibitor	10uM	121,6	92,9	103,8	**106,1**	36,4	36,4	48,9	**40,5**	**36,8**	-2
Temozolomide	Alkylating agent	10uM	29,9	33,0	28,1	**30,3**	15,6	22,6	18,0	**18,7**	**55,7**	-2
Oxaliplatin	Alkylating agent	10uM	9,4	7,4	10,1	**9,0**	10,2	9,1	11,3	**10,2**	**61,7**	-5
Sirolimus	mTOR-inhibitor	10uM	30,7	29,4	30,8	**30,3**	22,8	23,4	23,1	**23,1**	**56,2**	-6
Sorafenib	Kinase inhibitor	10uM	17,1	15,1	19,0	**17,1**	16,7	17,5	17,3	**17,2**	**55,7**	-8
Everolimus	mTOR-inhibitor	10uM	23,0	27,4	21,0	**23,8**	21,1	24,4	22,7	**22,7**	**61,7**	-8
5-azacytidine	Antimetabolite	10uM	44,6	41,3	34,8	**40,2**	35,5	41,4	38,1	**38,3**	**65,6**	-12
Dasatinib	Kinase inhibitor	10uM	21,6	17,4	19,8	**19,6**	22,7	22,2	19,5	**21,4**	**36,8**	-14
Estradiol	Hormone treatment	10uM	92,2	65,3	82,2	**79,9**	41,5	46,0	55,9	**47,8**	**36,8**	-18
Ruxolitinib	Kinase inhibitor	10uM	23,6	42,7	39,0	**35,1**	42,5	37,3	51,8	**43,8**	**65,6**	-21
Imatinib	Kinase inhibitor	10uM	73,1	43,6	81,5	**66,1**	56,2	79,5	63,1	**66,3**	**65,6**	-23
Vemurafenib	Kinase inhibitor	10uM	56,1	46,7	38,4	**47,0**	51,5	56,2	45,4	**51,0**	**56,2**	-25
Fulvestrant	Hormone treatment	10uM	90,9	74,7	42,2	**69,3**	56,3	40,7	57,0	**51,3**	**36,8**	-26
Verapamil	Calcium-channel blc	10uM	105,9	95,2	90,7	**97,2**	57,5	62,6	70,3	**63,5**	**36,8**	-28
Octreotide	Hormone treatment	10uM	108,4	92,4	105,5	**102,1**	29,7	34,1	31,8	**31,8**	**36,8**	6
Vincristine	Microtubule inhibit	10uM	11,9	9,9	11,1	**11,0**	3,0	3,0	3,1	**3,0**	**65,6**	4
Vinorelbine	Microtubule inhibit	10uM	7,2	9,2	11,8	**9,4**	3,6	2,9	3,4	**3,3**	**65,6**	3
Mitomycin	Anthracycline	10uM	3,2	3,3	2,9	**3,1**	2,3	2,0	2,0	**2,1**	**60,9**	0
Idarubicin	Anthracycline	10uM	1,0	1,0	0,9	**1,0**	0,6	0,9	0,8	**0,8**	**56,2**	0
Amsacrine	Acridine	10uM	0,7	0,9	0,8	**0,8**	0,6	0,8	0,8	**0,7**	**55,7**	0
Sunitinib	Kinase inhibitor	10uM	1,4	2,3	1,6	**1,8**	1,9	1,3	0,5	**1,2**	**55,7**	0
Daunorubicin	Anthracycline	10uM	0,6	0,8	0,7	**0,7**	1,2	0,6	0,6	**0,8**	**61,7**	0
Doxorubicin	Anthracycline	10uM	2,3	2,0	2,2	**2,2**	1,4	1,7	2,0	**1,7**	**61,7**	0
Vorinostat	HDAC inhibitor	10uM	4,5	5,1	4,1	**4,6**	2,8	3,6	2,5	**3,0**	**55,7**	0
Ponatinib	Kinase inhibitor	10uM	1,1	1,8	1,5	**1,5**	1,3	1,8	1,2	**1,5**	**65,6**	0
Epirubicin	Anthracycline	10uM	1,6	1,7	1,6	**1,6**	1,7	1,6	1,5	**1,6**	**61,7**	-1
Dactinomycin	Anthracycline	10uM	1,4	1,8	1,6	**1,6**	2,1	1,7	1,6	**1,8**	**55,7**	-1
Alectinib	Kinase inhibitor	10uM	1,1	0,1	0,0	**0,4**	1,5	1,4	1,3	**1,4**	**60,9**	-1
Gemcitabine	Antimetabolite	10uM	4,2	4,3	3,7	**4,1**	3,7	4,3	3,3	**3,7**	**56,2**	-1
Paclitaxel	Microtubule inhibit	10uM	13,4	14,2	13,3	**13,6**	9,1	10,1	10,6	**9,9**	**56,2**	-2
Mitoxantrone	Anthracycline	10uM	4,6	4,2	6,9	**5,2**	5,8	6,2	5,8	**6,0**	**60,9**	-3
Etoposide	Topoisomerase inhil	10uM	12,4	11,4	13,1	**12,3**	9,2	9,8	11,2	**10,1**	**56,2**	-3
Melphalan	Alkylating agent	10uM	18,0	14,4	15,9	**16,1**	11,8	13,7	15,0	**13,5**	**56,2**	-4
Irinotecan	Topoisomerase inhil	10uM	13,9	14,6	11,8	**13,5**	12,4	13,9	12,9	**13,0**	**61,7**	-5
Palbociclib	Kinase inhibitor	10uM	17,8	16,8	19,7	**18,1**	17,7	16,1	17,0	**16,9**	**65,6**	-5

Bendamustine	Alkylating agent	10uM	105,8	89,2	71,8	**88,9**	34,3	46,2	33,4	**37,9**	**36,8**	-5
Venetoclax	BCL-2 inhibitor	10uM	13,1	12,2	12,5	**12,6**	12,8	11,6	12,9	**12,4**	**55,7**	-5
Carboplatin	Alkylating agent	10uM	82,1	62,1	77,5	**73,9**	46,8	57,1	50,2	**51,4**	**61,7**	-6
Regorafenib	Kinase inhibitor	10uM	13,1	12,5	13,8	**13,1**	12,7	17,1	13,8	**14,5**	**61,7**	-6
Olaparib	PARP inhibitor	10uM	44,9	42,8	35,2	**41,0**	24,2	23,0	21,8	**23,0**	**36,8**	-8
Fluorouracil	Antimetabolite	10uM	16,6	22,5	18,9	**19,3**	20,4	19,5	20,5	**20,1**	**61,7**	-8
Tamoxifen	Hormone treatment	10uM	48,7	44,3	49,3	**47,4**	45,2	49,7	38,5	**44,5**	**60,9**	-16
Erlotinib	Kinase inhibitor	10uM	39,4	48,2	31,7	**39,8**	43,7	36,3	43,9	**41,3**	**56,2**	-19
Gefitinib	Kinase inhibitor	10uM	46,6	55,8	42,4	**48,2**	57,8	33,5	50,6	**47,3**	**56,2**	-20

Supplementary Data 2

Bliss Score vs viability in PBMC monocultures following treatment with conventional anticancer agents

HCT116-GFP		Bliss Score				Viability PBMC monoculture			
**Std treatments**	**Conc.**	**Rep 1**	**Rep 2**	**Rep 3**	**Avg**	**Rep 1**	**Rep 2**	**Rep 3**	**Avg**
Oxaliplatin	1uM	10,5	11,2	3,6	8,4	69,6	78,6	83,1	77,1
Oxaliplatin	10uM	-0,3	-1,9	-4,2	-2,1	19,2	22,2	22,0	21,1
Oxaliplatin	30uM	-4,3	-2,7	-4,8	-3,9	10,4	14,0	11,9	12,1
Busulfan	1uM	1,8	2,1	2,3	2,1	90,7	97,4	90,1	92,8
Busulfan	10uM	0,0	-1,6	-7,2	-2,9	75,7	80,8	92,3	82,9
Busulfan	30uM	5,2	-6,8	0,5	-0,4	68,5	79,4	90,8	79,6
Temozolomide	1uM	-5,3	3,5	1,0	-0,3	91,0	99,1	84,9	91,7
Temozolomide	10uM	4,4	-2,8	1,1	0,9	86,3	81,5	74,7	80,8
Temozolomide	30uM	2,0	-2,2	2,9	0,9	68,0	77,0	73,7	72,9
Thioguanine	1uM	18,2	24,4	14,8	19,1	59,0	48,2	47,7	51,7
Thioguanine	10uM	11,1	12,9	8,8	10,9	31,8	35,1	36,8	34,6
Thioguanine	30uM	0,5	-1,5	-2,4	-1,1	24,5	25,6	24,7	24,9
5-azacytidine	1uM	0,8	1,6	7,6	3,3	99,0	107,8	100,4	102,4
5-azacytidine	10uM	9,4	-0,9	3,9	4,1	47,6	67,9	58,8	58,1
5-azacytidine	30uM	2,0	-4,2	-1,1	-1,1	17,5	21,2	18,9	19,2
Dasatinib	1uM	-33,8	-31,0	-44,7	-36,5	10,5	10,2	11,2	10,6
Dasatinib	10uM	-12,7	-20,6	-27,5	-20,3	9,1	7,7	8,4	8,4
Dasatinib	30uM	-6,0	-9,4	-5,9	-7,1	3,3	3,4	3,6	3,5
Imatinib	1uM	-16,0	-7,6	-12,6	-12,1	89,0	107,9	103,6	100,1
Imatinib	10uM	-18,5	-20,4	-16,9	-18,6	20,7	36,2	30,9	29,3
Imatinib	30uM	-4,4	-4,3	-4,0	-4,2	2,4	2,1	2,2	2,2
Dexamethasone	1uM	-33,5	-32,7	-24,0	-30,1	43,9	56,8	57,2	52,6
Dexamethasone	10uM	-24,2	-34,7	-26,3	-28,4	42,5	65,4	49,4	52,5
Dexamethasone	30uM	-19,3	-30,2	-30,7	-26,7	32,0	46,2	44,4	40,9
Sorafenib	1uM	7,2	8,9	14,5	10,2	113,6	89,5	95,6	99,6
Sorafenib	10uM	-0,6	-0,2	0,1	-0,2	29,2	22,1	23,9	25,1
Sorafenib	30uM	-0,4	-0,6	-0,7	-0,6	1,5	0,6	1,1	1,1
Ruxolitinib	1uM	-23,3	-17,9	-31,3	-24,2	24,2	16,5	20,4	20,4
Ruxolitinib	10uM	-20,1	-16,3	-12,0	-16,2	17,3	11,4	13,6	14,1
Ruxolitinib	30uM	-4,7	-3,0	-3,0	-3,6	11,3	6,9	7,2	8,5
Vemurafenib	1uM	-14,3	-8,2	-7,8	-10,1	92,3	73,3	75,6	80,4
Vemurafenib	10uM	-6,7	-4,0	1,0	-3,3	25,9	22,1	24,8	24,3
Vemurafenib	30uM	-2,7	-3,2	-9,6	-5,2	6,6	2,9	3,6	4,4
Temsirolimus	1uM	-9,8	-8,5	-9,7	-9,3	43,9	35,7	38,8	39,4
Temsirolimus	10uM	-10,8	-5,8	-0,2	-5,6	17,4	19,0	16,9	17,7
Temsirolimus	30uM	-1,5	0,0	-0,6	-0,7	1,2	0,6	0,5	0,8
Everolimus	1uM	-8,2	-10,5	-1,9	-6,9	48,7	36,7	38,5	41,3
Everolimus	10uM	-7,3	-5,8	-7,5	-6,9	34,8	28,5	30,2	31,2
Everolimus	30uM	-0,3	-0,5	-0,6	-0,5	1,2	0,5	0,5	0,7
Sirolimus	1uM	-5,1	-13,4	-4,7	-7,7	52,0	37,0	37,9	42,3
Sirolimus	10uM	-12,8	-5,6	-8,0	-8,8	40,2	30,7	33,3	34,7
Sirolimus	30uM	-0,6	-0,5	-0,4	-0,5	1,5	0,6	0,9	1,0
Estradiol	1uM	-1,2	-5,1	-11,4	-5,9	116,3	101,6	94,5	104,1

Estradiol	10uM	-5,1	-8,6	3,0	-3,5	99,6	85,9	81,5	89,0
Estradiol	30uM	5,9	5,9	5,9	5,9	69,4	52,1	58,0	59,8
Fulvestrant	1uM	-4,8	-0,4	3,9	-0,4	108,3	96,5	85,7	96,8
Fulvestrant	10uM	2,8	6,9	14,6	8,1	65,2	60,2	56,2	60,5
Fulvestrant	30uM	10,2	7,6	9,0	8,9	60,0	59,9	51,8	57,3
Verapamil	1uM	-5,2	3,2	-6,8	-2,9	121,3	102,8	99,0	107,7
Verapamil	10uM	-12,5	-4,2	-2,6	-6,4	107,8	92,4	93,6	98,0
Verapamil	30uM	-0,6	0,3	-1,9	-0,7	32,3	36,4	25,0	31,2

**A549-NLR**			**Bliss Score**				**Viability PBMC monoculture**		
**Std treatments**	**Conc.**	**Rep 1**	**Rep 2**	**Rep 3**	**Avg**	**Rep 1**	**Rep 2**	**Rep 3**	**Avg**
Oxaliplatin	1uM	-6,2	-1,1	-4,8	-4,0	69,6	78,6	83,1	77,1
Oxaliplatin	10uM	-4,2	-3,9	-4,8	-4,3	19,2	22,2	22,0	21,1
Oxaliplatin	30uM	-4,6	-4,8	-4,2	-4,5	10,4	14,0	11,9	12,1
Busulfan	1uM	1,5	9,8	-5,2	2,0	90,7	97,4	90,1	92,8
Busulfan	10uM	1,3	4,6	-2,3	1,2	75,7	80,8	92,3	82,9
Busulfan	30uM	-11,4	-7,2	-10,6	-9,7	68,5	79,4	90,8	79,6
Temozolomide	1uM	-1,8	3,3	-7,6	-2,0	91,0	99,1	84,9	91,7
Temozolomide	10uM	-1,1	-1,9	-7,5	-3,5	86,3	81,5	74,7	80,8
Temozolomide	30uM	-17,6	-10,7	-12,7	-13,7	68,0	77,0	73,7	72,9
Thioguanine	1uM	-1,3	2,9	-0,7	0,3	59,0	48,2	47,7	51,7
Thioguanine	10uM	-7,4	-5,3	-15,6	-9,4	31,8	35,1	36,8	34,6
Thioguanine	30uM	-14,8	-13,5	-10,7	-13,0	24,5	25,6	24,7	24,9
5-azacytidine	1uM	-23,6	-14,9	-36,2	-24,9	99,0	107,8	100,4	102,4
5-azacytidine	10uM	-33,1	-35,2	-34,0	-34,1	47,6	67,9	58,8	58,1
5-azacytidine	30uM	-24,0	-25,0	-20,7	-23,2	17,5	21,2	18,9	19,2
Dasatinib	1uM	-24,7	-27,4	-23,0	-25,0	10,5	10,2	11,2	10,6
Dasatinib	10uM	-15,7	-21,2	-24,3	-20,4	9,1	7,7	8,4	8,4
Dasatinib	30uM	-5,3	-7,7	-5,1	-6,0	3,3	3,4	3,6	3,5
Imatinib	1uM	-12,9	-7,6	-10,7	-10,4	89,0	107,9	103,6	100,1
Imatinib	10uM	-41,8	-45,5	-60,3	-49,2	20,7	36,2	30,9	29,3
Imatinib	30uM	-11,7	-10,9	-8,9	-10,5	2,4	2,1	2,2	2,2
Dexamethasone	1uM	-15,2	-14,5	-16,9	-15,5	43,9	56,8	57,2	52,6
Dexamethasone	10uM	-12,3	-8,4	-14,6	-11,8	42,5	65,4	49,4	52,5
Dexamethasone	30uM	-21,8	-20,0	-14,1	-18,6	32,0	46,2	44,4	40,9
Sorafenib	1uM	-0,9	-2,0	1,5	-0,5	113,6	89,5	95,6	99,6
Sorafenib	10uM	-6,4	-7,5	-4,7	-6,2	29,2	22,1	23,9	25,1
Sorafenib	30uM	-0,5	-1,3	-0,1	-0,6	1,5	0,6	1,1	1,1
Ruxolitinib	1uM	-51,3	-72,5	-42,6	-55,5	24,2	16,5	20,4	20,4
Ruxolitinib	10uM	-24,3	-27,9	-25,9	-26,0	17,3	11,4	13,6	14,1
Ruxolitinib	30uM	-7,3	-8,1	-6,4	-7,3	11,3	6,9	7,2	8,5
Vemurafenib	1uM	-13,9	-24,9	-28,4	-22,4	92,3	73,3	75,6	80,4
Vemurafenib	10uM	-34,6	-30,6	-21,9	-29,0	25,9	22,1	24,8	24,3
Vemurafenib	30uM	-10,8	-15,6	-8,4	-11,6	6,6	2,9	3,6	4,4
Temsirolimus	1uM	-8,9	-12,5	-9,2	-10,2	43,9	35,7	38,8	39,4
Temsirolimus	10uM	-9,7	-10,5	-4,4	-8,2	17,4	19,0	16,9	17,7
Temsirolimus	30uM	-0,2	1,0	-0,3	0,2	1,2	0,6	0,5	0,8
Everolimus	1uM	-9,7	-15,8	-5,3	-10,3	48,7	36,7	38,5	41,3

Everolimus	10uM	-11,5	-14,9	-14,7	-13,7	34,8	28,5	30,2	31,2
Everolimus	30uM	-3,5	-0,3	-1,6	-1,8	1,2	0,5	0,5	0,7
Sirolimus	1uM	-8,2	-12,5	-5,2	-8,6	52,0	37,0	37,9	42,3
Sirolimus	10uM	-14,0	-17,4	-12,2	-14,5	40,2	30,7	33,3	34,7
Sirolimus	30uM	-2,8	-1,3	-1,2	-1,8	1,5	0,6	0,9	1,0
Estradiol	1uM	-6,0	-14,3	-7,7	-9,4	116,3	101,6	94,5	104,1
Estradiol	10uM	-19,5	-11,2	-6,3	-12,3	99,6	85,9	81,5	89,0
Estradiol	30uM	-7,0	-9,3	-2,8	-6,3	69,4	52,1	58,0	59,8
Fulvestrant	1uM	-13,3	-21,1	-17,0	-17,1	108,3	96,5	85,7	96,8
Fulvestrant	10uM	-18,5	-18,4	-10,8	-15,9	65,2	60,2	56,2	60,5
Fulvestrant	30uM	-16,2	-18,7	-8,3	-14,4	60,0	59,9	51,8	57,3
Verapamil	1uM	-11,0	-9,8	-5,7	-8,8	121,3	102,8	99,0	107,7
Verapamil	10uM	-14,6	-4,4	-15,0	-11,3	107,8	92,4	93,6	98,0
Verapamil	30uM	-28,4	-31,4	-21,4	-27,1	32,3	36,4	25,0	31,2

Supplementary Data 3

Bliss Score vs viability in PBMC monocultures following treatment with concomitant drugs

HCT116-GFP		Bliss Score				Viability PBMC monoculture			
**Concomitant drug**	**Conc.**	**Rep 1**	**Rep 2**	**Rep 3**	**Avg**	**Rep 1**	**Rep 2**	**Rep 3**	**Avg**
Paracetamol	1uM	-4,2	-8,0	3,2	**-3,0**	83,7	92,5	109,2	**95,1**
Ibuprofen	1uM	-0,4	-5,2	2,5	**-1,0**	91,9	99,8	119,5	**103,7**
Acetylsalicylsyra	1uM	-5,2	-4,6	0,2	**-3,2**	92,8	92,6	110,2	**98,5**
Celecoxib	1uM	-3,8	-1,4	2,8	**-0,8**	84,8	97,6	112,5	**98,3**
Simvastatin	1uM	11,4	8,1	17,5	**12,3**	70,9	86,9	113,6	**90,5**
Mevastatin	1uM	7,1	6,5	7,8	**7,1**	96,9	116,7	127,8	**113,8**
Metformin	1uM	-2,4	1,1	3,5	**0,7**	109,0	100,3	120,2	**109,9**
Enalapril	1uM	-0,8	0,2	7,9	**2,4**	101,4	110,7	118,0	**110,0**
Metoprolol	1uM	1,3	-5,8	13,5	**3,0**	108,1	107,5	118,0	**111,2**
Metoclopramide	1uM	-2,1	2,2	1,3	**0,4**	119,0	118,3	126,1	**121,1**
Betamethasone	1uM	-37,5	-45,2	-28,4	**-37,0**	65,1	63,4	68,8	**65,8**
Prednisolone	1uM	-24,8	-46,1	-25,7	**-32,2**	65,3	51,8	53,9	**57,0**
Doxazosine	1uM	-5,0	-3,7	1,1	**-2,5**	87,2	89,3	98,4	**91,6**
Loratadine	1uM	-5,9	-13,7	-1,1	**-6,9**	83,8	88,2	88,3	**86,8**
Mycophenolate	1uM	-0,3	-1,4	2,7	**0,3**	36,0	44,4	47,7	**42,7**
Amoxicillin	1uM	-6,8	-7,9	-2,1	**-5,6**	109,7	99,6	100,9	**103,4**
Piperacillin	1uM	0,8	-7,7	-4,0	**-3,6**	98,6	108,1	111,3	**106,0**
Tazobactam	1uM	0,3	-5,7	7,1	**0,6**	93,5	99,9	102,8	**98,7**
Sertraline	1uM	-8,4	-14,4	-6,6	**-9,8**	95,3	90,5	107,6	**97,8**
Haloperidol	1uM	-4,3	-17,4	-6,8	**-9,5**	105,2	104,7	105,0	**105,0**
Morphine	1uM	-3,4	-1,8	-3,7	**-3,0**	112,5	99,6	106,0	**106,0**
Omeprazol	1uM	-5,5	-14,7	-4,5	**-8,2**	98,2	100,4	102,6	**100,4**
Paracetamol	10uM	-1,1	3,5	-10,7	**-2,8**	83,4	100,3	117,0	**100,2**
Ibuprofen	10uM	4,7	10,4	-5,0	**3,4**	86,8	77,3	124,2	**96,1**
Acetylsalicylsyra	10uM	-0,5	8,6	-1,5	**2,2**	73,3	88,4	112,6	**91,4**
Celecoxib	10uM	4,0	7,8	-0,4	**3,8**	74,5	88,0	75,8	**79,5**
Simvastatin	10uM	14,8	24,6	18,9	**19,4**	33,6	39,9	41,8	**38,4**
Mevastatin	10uM	21,8	29,4	18,3	**23,2**	42,1	54,9	61,6	**52,8**
Metformin	10uM	1,0	14,1	-10,5	**1,5**	108,8	111,5	115,9	**112,0**
Enalapril	10uM	-0,6	5,1	-4,6	**0,0**	83,1	103,3	124,4	**103,6**
Metoprolol	10uM	-3,2	4,9	-2,2	**-0,2**	115,1	100,7	116,6	**110,8**
Metoclopramide	10uM	-4,7	0,8	-2,8	**-2,2**	127,8	133,3	135,3	**132,1**
Betamethasone	10uM	-37,6	-34,7	-25,3	**-32,5**	58,7	67,5	73,0	**66,4**
Prednisolone	10uM	-28,4	-42,4	-29,8	**-33,5**	56,4	49,5	53,3	**53,1**
Doxazosine	10uM	10,3	0,3	4,6	**5,1**	67,8	68,7	83,2	**73,2**
Loratadine	10uM	1,8	-5,5	-5,1	**-2,9**	42,3	57,1	73,5	**57,6**
Mycophenolate	10uM	-2,4	-2,8	-1,5	**-2,2**	27,8	34,0	39,0	**33,6**
Amoxicillin	10uM	-4,2	-4,6	-2,3	**-3,7**	103,1	93,0	99,6	**98,6**
Piperacillin	10uM	1,7	-8,8	-1,1	**-2,8**	92,2	92,5	108,0	**97,6**
Tazobactam	10uM	-1,5	-3,6	-11,4	**-5,5**	92,6	86,7	98,2	**92,5**
Sertraline	10uM	-23,0	-10,1	-2,7	**-11,9**	1,2	1,5	1,9	**1,5**
Haloperidol	10uM	-5,5	-10,6	-7,0	**-7,7**	83,5	81,8	88,3	**84,5**
Morphine	10uM	-3,8	-8,1	-3,3	**-5,1**	111,4	107,7	114,5	**111,2**

Omeprazol	10uM	-10,5	-4,6	3,8	**-3,8**	96,3	96,8	105,4	**99,5**
Paracetamol	30uM	0,5	8,3	9,0	**5,9**	74,8	98,3	123,0	**98,7**
Ibuprofen	30uM	-1,7	-7,2	-1,6	**-3,5**	93,7	85,3	112,7	**97,2**
Acetylsalicylsyra	30uM	-2,9	7,2	1,7	**2,0**	92,1	105,3	119,7	**105,7**
Celecoxib	30uM	0,3	11,0	9,4	**6,9**	59,8	79,6	83,7	**74,3**
Simvastatin	30uM	5,9	8,0	6,2	**6,7**	23,9	24,9	25,2	**24,6**
Mevastatin	30uM	16,3	16,9	15,9	**16,4**	34,9	39,8	45,0	**39,9**
Metformin	30uM	-3,3	5,0	4,5	**2,0**	92,4	119,6	122,7	**111,5**
Enalapril	30uM	-7,8	16,9	7,3	**5,4**	83,2	105,0	119,9	**102,7**
Metoprolol	30uM	1,2	3,9	-1,4	**1,2**	101,9	119,5	130,3	**117,2**
Metoclopramide	30uM	-9,0	8,3	4,2	**1,2**	130,3	129,4	125,1	**128,3**
Betamethasone	30uM	-26,4	-35,9	-24,8	**-29,0**	49,5	53,5	55,7	**52,9**
Prednisolone	30uM	-26,0	-38,3	-25,8	**-30,0**	45,6	39,6	46,0	**43,7**
Doxazosine	30uM	0,6	0,3	1,5	**0,8**	1,3	1,8	1,7	**1,6**
Loratadine	30uM	5,0	5,4	5,7	**5,4**	17,2	26,0	28,1	**23,8**
Mycophenolate	30uM	-1,3	-1,8	-1,0	**-1,3**	30,1	34,2	39,0	**34,5**
Amoxicillin	30uM	-0,1	-7,4	0,7	**-2,3**	100,8	93,8	109,9	**101,5**
Piperacillin	30uM	-0,3	-6,2	-5,8	**-4,1**	90,7	88,1	101,8	**93,5**
Tazobactam	30uM	-3,0	8,4	3,2	**2,8**	98,5	90,3	107,4	**98,7**
Sertraline	30uM	-0,1	-0,1	-0,1	**-0,1**	0,6	0,9	0,9	**0,8**
Haloperidol	30uM	-1,5	0,1	10,4	**3,0**	22,3	16,6	19,5	**19,5**
Morphine	30uM	-6,6	-1,4	-3,4	**-3,8**	106,6	104,6	100,7	**104,0**
Omeprazol	30uM	-3,0	-5,1	0,5	**-2,5**	82,7	81,8	94,6	**86,4**

**A549-NLR**			**Bliss Score**				**Viability PBMC monoculture**		
**Concomitant drug**	**Conc.**	**Rep 1**	**Rep 2**	**Rep 3**	**Avg**	**Rep 1**	**Rep 2**	**Rep 3**	**Avg**
Paracetamol	1uM	-16,1	-3,4	-6,6	**-8,7**	83,7	92,5	109,2	**95,1**
Ibuprofen	1uM	-5,1	-0,2	-5,3	**-3,5**	91,9	99,8	119,5	**103,7**
Acetylsalicylsyra	1uM	-9,9	-4,1	-5,9	**-6,6**	92,8	92,6	110,2	**98,5**
Celecoxib	1uM	-10,3	-3,4	-3,0	**-5,6**	84,8	97,6	112,5	**98,3**
Simvastatin	1uM	-1,8	0,3	-3,0	**-1,5**	70,9	86,9	113,6	**90,5**
Mevastatin	1uM	1,5	-1,9	-9,3	**-3,2**	96,9	116,7	127,8	**113,8**
Metformin	1uM	-0,8	3,9	-4,9	**-0,6**	109,0	100,3	120,2	**109,9**
Enalapril	1uM	-4,4	0,1	-6,8	**-3,7**	101,4	110,7	118,0	**110,0**
Metoprolol	1uM	-4,8	-3,6	-0,7	**-3,0**	108,1	107,5	118,0	**111,2**
Metoclopramide	1uM	-8,7	-1,2	-13,5	**-7,8**	119,0	118,3	126,1	**121,1**
Betamethasone	1uM	-12,9	-12,2	-19,1	**-14,7**	65,1	63,4	68,8	**65,8**
Prednisolone	1uM	-12,1	-7,0	-17,5	**-12,2**	65,3	51,8	53,9	**57,0**
Doxazosine	1uM	-14,6	4,1	-8,8	**-6,4**	87,2	89,3	98,4	**91,6**
Loratadine	1uM	-10,0	5,1	-9,2	**-4,7**	83,8	88,2	88,3	**86,8**
Mycophenolate	1uM	-12,4	-5,6	-11,3	**-9,8**	36,0	44,4	47,7	**42,7**
Amoxicillin	1uM	-8,2	2,6	1,8	**-1,3**	109,7	99,6	100,9	**103,4**
Piperacillin	1uM	-12,3	-3,3	-7,3	**-7,6**	98,6	108,1	111,3	**106,0**
Tazobactam	1uM	-4,9	3,6	-4,8	**-2,0**	93,5	99,9	102,8	**98,7**
Sertraline	1uM	-1,2	8,2	3,0	**3,3**	95,3	90,5	107,6	**97,8**
Haloperidol	1uM	-10,3	7,7	-0,6	**-1,1**	105,2	104,7	105,0	**105,0**
Morphine	1uM	-10,0	-0,3	-7,3	**-5,9**	112,5	99,6	106,0	**106,0**
Omeprazol	1uM	-1,0	-6,2	-3,3	**-3,5**	98,2	100,4	102,6	**100,4**

Paracetamol	10uM	-4,4	-4,2	-2,8	**-3,8**	83,4	100,3	117,0	**100,2**
Ibuprofen	10uM	-7,2	-6,2	-1,7	**-5,0**	86,8	77,3	124,2	**96,1**
Acetylsalicylsyra	10uM	-6,4	-0,7	-3,7	**-3,6**	73,3	88,4	112,6	**91,4**
Celecoxib	10uM	-15,1	-6,9	-14,4	**-12,2**	74,5	88,0	75,8	**79,5**
Simvastatin	10uM	-3,9	-2,0	-3,3	**-3,1**	33,6	39,9	41,8	**38,4**
Mevastatin	10uM	-3,2	-2,0	-3,6	**-2,9**	42,1	54,9	61,6	**52,8**
Metformin	10uM	-5,0	-5,9	-0,7	**-3,9**	108,8	111,5	115,9	**112,0**
Enalapril	10uM	-7,1	-3,3	-4,7	**-5,0**	83,1	103,3	124,4	**103,6**
Metoprolol	10uM	-3,9	5,1	4,2	**1,8**	115,1	100,7	116,6	**110,8**
Metoclopramide	10uM	-10,1	-4,4	-6,3	**-6,9**	127,8	133,3	135,3	**132,1**
Betamethasone	10uM	-11,3	-10,9	-14,8	**-12,3**	58,7	67,5	73,0	**66,4**
Prednisolone	10uM	-10,8	-10,3	-13,3	**-11,5**	56,4	49,5	53,3	**53,1**
Doxazosine	10uM	-17,4	-28,1	-30,2	**-25,3**	67,8	68,7	83,2	**73,2**
Loratadine	10uM	-26,6	-21,5	-33,8	**-27,3**	42,3	57,1	73,5	**57,6**
Mycophenolate	10uM	-6,7	-8,1	-8,7	**-7,9**	27,8	34,0	39,0	**33,6**
Amoxicillin	10uM	-5,5	-2,2	-5,8	**-4,5**	103,1	93,0	99,6	**98,6**
Piperacillin	10uM	-5,9	-2,0	-6,3	**-4,7**	92,2	92,5	108,0	**97,6**
Tazobactam	10uM	-7,9	-0,5	-11,1	**-6,5**	92,6	86,7	98,2	**92,5**
Sertraline	10uM	-18,9	-24,7	-25,6	**-23,1**	1,2	1,5	1,9	**1,5**
Haloperidol	10uM	-6,1	-6,6	-5,2	**-6,0**	83,5	81,8	88,3	**84,5**
Morphine	10uM	-8,8	-12,1	-1,3	**-7,4**	111,4	107,7	114,5	**111,2**
Omeprazol	10uM	-9,1	-8,5	-18,7	**-12,1**	96,3	96,8	105,4	**99,5**
Paracetamol	30uM	-13,4	-6,4	-7,0	**-8,9**	74,8	98,3	123,0	**98,7**
Ibuprofen	30uM	-14,8	-9,5	-7,3	**-10,5**	93,7	85,3	112,7	**97,2**
Acetylsalicylsyra	30uM	-1,5	-5,4	-1,4	**-2,8**	92,1	105,3	119,7	**105,7**
Celecoxib	30uM	-20,3	-20,0	-18,3	**-19,5**	59,8	79,6	83,7	**74,3**
Simvastatin	30uM	-0,9	-0,7	-1,4	**-1,0**	23,9	24,9	25,2	**24,6**
Mevastatin	30uM	-2,4	-2,9	-2,2	**-2,5**	34,9	39,8	45,0	**39,9**
Metformin	30uM	-2,2	-7,2	-0,6	**-3,3**	92,4	119,6	122,7	**111,5**
Enalapril	30uM	-7,8	-7,5	-8,8	**-8,0**	83,2	105,0	119,9	**102,7**
Metoprolol	30uM	-3,3	-4,2	4,1	**-1,1**	101,9	119,5	130,3	**117,2**
Metoclopramide	30uM	-6,5	-11,1	-6,0	**-7,9**	130,3	129,4	125,1	**128,3**
Betamethasone	30uM	-14,5	-12,7	-11,2	**-12,8**	49,5	53,5	55,7	**52,9**
Prednisolone	30uM	-14,1	-14,4	-21,2	**-16,6**	45,6	39,6	46,0	**43,7**
Doxazosine	30uM	-1,6	-1,9	-1,6	**-1,7**	1,3	1,8	1,7	**1,6**
Loratadine	30uM	-6,5	-3,7	-8,0	**-6,1**	17,2	26,0	28,1	**23,8**
Mycophenolate	30uM	-6,5	-7,1	-7,9	**-7,2**	30,1	34,2	39,0	**34,5**
Amoxicillin	30uM	-3,6	-10,0	-8,0	**-7,2**	100,8	93,8	109,9	**101,5**
Piperacillin	30uM	-8,4	-5,5	-11,4	**-8,4**	90,7	88,1	101,8	**93,5**
Tazobactam	30uM	-7,8	-6,2	-9,5	**-7,8**	98,5	90,3	107,4	**98,7**
Sertraline	30uM	0,0	-0,1	-0,1	**-0,1**	0,6	0,9	0,9	**0,8**
Haloperidol	30uM	-25,3	-25,1	-18,5	**-23,0**	22,3	16,6	19,5	**19,5**
Morphine	30uM	-11,7	1,9	-9,4	**-6,4**	106,6	104,6	100,7	**104,0**
Omeprazol	30uM	-12,9	-15,3	-18,2	**-15,5**	82,7	81,8	94,6	**86,4**
